# Targeting ferroptosis: a promising avenue for ovarian cancer treatment

**DOI:** 10.3389/fimmu.2025.1578723

**Published:** 2025-06-05

**Authors:** Xiaolan Wu, Qizhi Liu, Zhili Jiang, Guiyun Wang, Lingyu Liao, Xiaojuan Ye, Min Xing, Han Sun, Qiying Liu, Huiping Liu

**Affiliations:** ^1^ School of Integrated Chinese and Western Medicine, Hunan University of Chinese Medicine, Changsha, Hunan, China; ^2^ Hunan Provincial Key Laboratory of Traditional Chinese Medicine Prescription and Syndrome Differentiation Translational Medicine, Hunan University of Chinese Medicine, Changsha, Hunan, China; ^3^ Hunan University of Chinese Medicine, Key Laboratory of Traditional Chinese Medicine Oncology of Hunan Province, Changsha, Hunan, China; ^4^ Department of Gynaecology, The First Hospital of Hunan University of Chinese Medicine, Changsha, Hunan, China; ^5^ College of Traditional Chinese Medicine, Hunan University of Chinese Medicine, Changsha, Hunan, China; ^6^ Medical School, Hunan University of Chinese Medicine, Changsha, Hunan, China; ^7^ Department of Traditional Chinese Medicine, Changsha Hospital for Maternal and Child Health Care, Changsha, Hunan, China

**Keywords:** OC, ferroptosis, lipid peroxidation, drug resistance, targeted therapy

## Abstract

Ovarian cancer(OC) is the second most common gynecological malignancy worldwide. While traditional treatments such as cytoreductive surgery, chemotherapy, and targeted drugs have made progress, patients with advanced disease still face high recurrence rates and resistance to treatment. As a result, there is an urgent need to develop new therapeutic strategies. Ferroptosis, a novel form of programmed cell death characterized by iron-dependent lipid peroxidation, has recently gained attention for its potential in cancer therapy. Studies indicate that OC cells are highly sensitive to ferroptosis, and targeting this pathway can effectively overcome chemotherapy resistance and improve treatment outcomes. This review systematically examines the molecular mechanisms of ferroptosis and its role in OC, with a focus on its involvement in tumor initiation, progression, TME and resistance. Furthermore, we highlight the research advancements on various ferroptosis inducers, including natural products, small molecule compounds, and nanotechnology, and explore their potential in overcoming resistance and enhancing patient prognosis. We also discuss the challenges facing ferroptosis-based treatments for OC, such as species differences, drug resistance, personalized treatment needs, and clinical translation issues. Ultimately, targeted modulation of ferroptosis offers new hope for OC therapy. Future research should focus on further elucidating its molecular mechanisms and exploring effective inducers and combination therapies to enhance its clinical applicability in precision and personalized medicine.

## 1 Introduction

Ovarian cancer(OC) ranks as the second most common malignant tumor among gynecological cancers worldwide, accounting for an estimated 3.7% of cases and 4.7% of cancer deaths in 2020 (1). OC is a highly heterogeneous disease, with only about half of patients surviving 5 years after diagnosis (2). Although cytoreductive surgery, taxane-based platinum chemotherapy, and targeted therapies—such as bevacizumab and PARP inhibitors— have made significant progress in clinical settings, the majority of patients with advanced stages still experience disease recurrence and treatment failure (3). Consequently, the prognosis for patients with advanced OC remains poor, characterized by high relapse rates and resistance to conventional therapies (4).This highlights the urgent need for novel therapeutic strategies, particularly those targeting resistance mechanisms, to improve survival outcomes (5).

In recent years, ferroptosis has attracted widespread attention as a type of programmed cell death. First proposed by Dixon et al. in 2012, ferroptosis differs from other cell death pathways, such as apoptosis and necrosis, and is characterized by the accumulation of iron-dependent lipid peroxides (6).This process is driven by factors including intracellular iron overload, glutathione(GSH) depletion, and excessive reactive oxygen species (ROS)generation, which ultimately result in irreversible damage to the cell membrane and cell death (7). Given its unique regulation of cancer cell metabolic reprogramming and iron metabolism, ferroptosis plays a crucial role in tumors and has emerged as a hot topic in anti-cancer research (8–10).OC cells exhibit a high dependence on iron metabolism, making them significantly more susceptible to ferroptosis compared to normal cells (11). Studies have demonstrated that activating ferroptosis can inhibit OC growth and reverse chemotherapy resistance (12, 13). Particularly in chemotherapy-resistant OC, ferroptosis can selectively target tumor cells while sparing normal tissues by modulating redox homeostasis and lipid metabolism (14–16). As a result, targeting ferroptosis offers a promising strategy to overcome chemotherapy resistance and improve the prognosis of advanced OC patients (17–21). However, despite significant progress in understanding the molecular mechanisms and regulatory networks of ferroptosis, its clinical application in treating OC still faces challenges, including the need for efficient and selective induction of ferroptosis and the integration of existing therapies to enhance treatment efficacy.

This article reviews the research progress on targeted ferroptosis in OC treatment, with a focus on the molecular mechanisms of ferroptosis, its role in treating OC, the relationship between chemoresistance and ferroptosis, and the potential of natural products and small molecule drugs in inducing ferroptosis. Additionally, the article explores the potential of natural products and small molecule drugs in inducing ferroptosis and the potential applications of emerging nanotechnologies and combination treatment strategies. It also addresses the limitations of current research and offers insights for future studies on targeted ferroptosis in OC therapy.

## 2 Molecular mechanisms of ferroptosis

### 2.1 Overview of ferroptosis

The term ferroptosis is derived from the Latin word ferrum, meaning iron. Research on lipid metabolism, oxidative stress, and iron homeostasis forms the foundation for understanding ferroptosis. In 1876, Henry John Horstman Fenton proposed the Fenton reaction. Iron, a key trigger and mediator of ferroptosis signaling, can catalyze the formation of harmful free radicals through the Fenton reaction (22). In 1929, polyunsaturated fatty acids (PUFAs), such as linoleic acid and linolenic acid, were recognized as crucial substrates for lipid peroxidation in ferroptosis (23). Phospholipids (PLs), which are integral components of cell membranes, contain PUFA groups and play a critical role in the process of ferroptosis (24).In 1955, Harry Eagle and colleagues discovered that cysteine (Cys) is essential for cell survival, and its deficiency, leading to GSH depletion, can result in cell death (25). In 1985, Helmut Sies coined the term “oxidative stress” (26), referring to a process where an excess of oxidizing substances—such as free radicals and peroxides—disrupts intracellular redox balance, triggering a range of harmful biological effects (27). GSH depletion impairs the cell’s ability to counteract oxidative stress, making it more susceptible to damage from oxidants like hydrogen peroxide (28). Regarding lipid metabolism, several studies have identified key mechanisms involved in lipid metabolism during ferroptosis (23, 29). These foundational studies provide key insights into the mechanisms driving ferroptosis.

Since 2012, ferroptosis has emerged as a prominent topic in biomedical research, with a rapid increase in related studies (30, 31). It has been implicated in a wide range of biological processes, including development, aging, immunity, and cancer. Over the past decade, significant breakthroughs have been made in understanding the mechanisms of ferroptosis and harnessing it for therapeutic benefits.

### 2.2 Regulation of ferroptotic signaling

#### 2.2.1 Classical ferroptotic pathway

##### 2.2.1.1 GPX4 and the GSH-dependent antioxidant system

Glutathione peroxidase4 (GPX4) is a key molecule in inhibiting ferroptosis, primarily by preventing the propagation of the lipid peroxidation chain reaction through the reduction of lipid hydroperoxide (LOOH) to inactive LOH(phospholipid alcohols). The activity of GPX4 relies on the tripeptide GSH as a reducing agent, and GSH synthesis is central to its function. This synthesis is driven by two enzymatic reactions: Glutamate-Cysteine Ligase (GCL) and Glutathione Synthetase(GSS).GCL catalyzes the binding of glutamate to cysteine, while GSS facilitates the attachment of glycine to glutamylcysteine, forming GSH. To support GSH synthesis, cells uptake cysteine from the extracellular environment via the system Xc-, which consists of the Solute Carrier Family 7 Member 11(SLC7A11) and Solute Carrier Family 3 Member 2 (SLC3A2) subunits. Inhibition of GPX4 disrupts intracellular iron homeostasis and lipid peroxide reduction, inducing ferroptosis (32).

##### 2.1.1.2 Lipid peroxidation and PUFA

Lipid peroxidation is a key determinant of ferroptosis sensitivity. It is a biochemical process in which free radicals, such as ROS and reactive nitrogen species (RNS), attack and oxidize lipids in cell membranes and organelles. During lipid peroxidation, ROS and RNS react with PUFAs in plasma and organelle membranes to generate lipid peroxides (33). ROS are oxygen-containing, chemically reactive molecules that can initiate or amplify ferroptosis sensitivity in various cell types and tissues (34). RNS, which are reactive chemical species derived from nitrogen sources, include peroxynitrite, nitrosyl radicals and nitroxides. Studies have shown that ferroptosis can be induced by modulating cellular lipid peroxidation using peroxynitrite generators (35).

The mechanism of the lipid peroxidation chain reaction involves three stages: initiation, propagation, and termination, along with various regulatory mechanisms and key molecules. In the initiation stage, promoters such as hydroxyl radicals remove allylic hydrogen atoms, generating lipid radicals with a carbon core. During propagation, these lipid radicals rapidly combine with oxygen to form lipid peroxyl radicals. This stage is driven by divalent iron (Fe²^+^), which reacts with hydrogen peroxide through the Fenton reaction to produce highly reactive hydroxyl (•OH) radicals. These •OH radicals can directly attack PUFAs, such as linoleic acid and arachidonic acid, in cell membranes. Due to their multiple double bonds, these fatty acids are highly susceptible to reaction with free radicals, resulting in lipid peroxide formation. The accumulation of iron ions exacerbates lipid peroxidation, particularly in PUFAs within cell membranes. In addition to Fenton-type chemistry, enzymes like lipoxygenases and P450(Cytochrome P450) oxidoreductases can also promote lipid peroxidation in membranes. Once initiated, the lipid peroxidation chain reaction accelerates, with lipid peroxides such as LOOH further cleaving to produce new free radicals, like peroxyl radicals, which attack other lipid molecules and perpetuate the chain reaction.

The termination phase of lipid peroxidation relies on antioxidant systems, such as Ferroptosis Suppressor Protein 1(FSP1), vitamin E, and free radical scavengers, to neutralize free radicals and inhibit ferroptosis. FSP1 reduces coenzyme Q10(CoQ10) to its reduced form, coenzyme QH_2_, using nicotinamide adenine dinucleotide phosphate (NADPH). Vitamin E reacts with lipid peroxyl radicals, forming a non-radical product and preventing further radical propagation. Free radical scavengers, such as reactive antioxidants (RTAs), block the lipid peroxidation chain reaction by directly interacting with free radicals. The lipid peroxidation chain reaction continues until these antioxidant systems intervene and the termination product is formed (36). Exogenous supplementation of Monounsaturated Fatty Acid(MUFA), stearoyl-CoA desaturase (SCD1)-mediated cellular MUFA production, and Acyl-CoA Synthetase Long-Chain Family Member 3 (ACSL3)-dependent MUFA membrane enrichment have been reported to reduce the propensity of cells to die by ferroptosis (37).The main byproduct of lipid peroxidation is lipid hydroperoxides. Lipid peroxidation produces a variety of aldehydes, including as Malondialdehyde (MDA), propionaldehyde, hexanal, and 4-HNE. PLs are the major lipid structures involved in ferroptosis, and lipid peroxidation of the phospholipid bilayer can promote ferroptosis. Among the fatty acids in PLs, PUFAs are the most susceptible to autoxidation—a free radical chain reaction that converts lipids into lipid hydroperoxides (38). Phospholipid hydroperoxides (PLOOH) are the key executors of ferroptosis. PLs containing two PUFA tails are particularly potent drivers of this process (39). PL-PUFA_2_, rather than PL-PUFA_1_, represents the key lipid class involved in ferroptosis (40).

PLs containing PUFAs are the primary substrates for lipid peroxidation in ferroptosis and are upregulated by enzymes such as acyl-CoA synthetase long-chain family member 4 (ACSL4), lysophosphatidylcholine acyltransferase 3 (LPCAT3), ALOXs, and Cytochrome P450 oxidoreductase(POR) (41).The membrane remodeling enzymes, ACSL4 (42) and LPCAT3, are key drivers of ferroptosis. ACSL4 enhances ferroptosis by promoting lipid oxidation. Interestingly, in certain cell types, ACSL4 may also play a protective role against ferroptosis (43). This may be related to the metabolic state of cells and the tumor microenvironment. LPCAT3 is transcriptionally regulated by the YAP/ZEB/EP300 axis and works in conjunction with ACSL4 and YAP to modulate ferroptosis sensitivity (44). Lipoxygenase (LOX) is a dioxygenase that catalyzes the conversion of polyunsaturated fatty acids, such as linoleic acid and arachidonic acid, into their corresponding hydroperoxides (45). POR is an enzyme essential for various metabolic processes (46) and plays a role in initiating lipid peroxidation. Studies have shown that POR can induce ferroptosis by promoting the peroxidation of membrane polyunsaturated PLs (47).

Guanine cyclohydrolase 1 (GCH1) is the rate-limiting enzyme in the tetrahydrobiopterin (BH_4_) biosynthesis pathway. BH_4_ is not only a crucial cofactor for various enzymes, such as nitric oxide synthase, but also plays a key role in ferroptosis defense by inhibiting lipid peroxidation. Studies have shown that BH_4_ can protect against ferroptosis by directly scavenging lipid peroxides and inhibiting the generation of ROS (48). In addition, GCH1 and BH4 can also support the regeneration of coenzyme Q, working synergistically with the FSP1 system to protect cells from lipid oxidative damage (49).

##### 2.1.1.3 Iron metabolism and the Fenton reaction

Ferroptosis is an iron-dependent form of cell death closely associated with disorders of iron metabolism (50). Iron is both a catalyst and a key regulator of ferroptosis. As an essential mineral, it is involved in numerous physiological processes. Fe²^+^ from heme and non-heme sources is transported to the basal surface of enterocytes, reoxidized to Fe³^+^ by ferroferrin (HEPH), exported via the export protein ferroportin (FPN) (51), and then loaded onto the circulating iron transporter transferrin (TF) for systemic delivery. TF-bound iron is transported to peripheral cells, where it binds to transferrin receptor 1 (TFR1). The binding activates the non-receptor tyrosine kinase Src, which, in turn, triggers the endocytosis of the TF-TFR1 complex (52). Once inside the cell, Fe³^+^ is released and reduced to Fe²^+^ by the six-transmembrane epithelial antigen of the prostate (STEAP) family reductase (53). This Fe²^+^ participates in DNA synthesis, cellular respiration, and energy metabolism. Iron can also be stored in ferritin or exported to the circulation via FPN (54). A portion of Fe²^+^ is transported from endosomes to the cytosol by Divalent metal transporter 1(DMT1), becoming part of the metabolically active labile iron pool (LIP).

The first step of ferroptosis is the excessive accumulation of intracellular iron ions. Increased iron intake due to diet, environmental factors, or other pathological conditions disrupts systemic iron homeostasis. This leads to enhanced TfR-mediated iron influx, while iron storage proteins, such as ferritin, become saturated and can no longer store excess iron. As a result, free iron (Fe²^+^) levels rise, contributing to ferroptosis. The liver regulates iron absorption, recycling, and tissue levels by secreting hepcidin, which reduces iron efflux by negatively regulating FPN (55). Increased hepcidin secretion binds to FPN and triggers its degradation (55). Iron regulatory proteins (IRPs) bind to the 5’ IRE of FPN1 and FT mRNAs, resulting in translational repression (56). Additionally, IRPs bind to the 3’ IRE of TFR1 mRNA, stabilizing it and enhancing TFR1 protein synthesis. As a consequence, TFR1 levels increase while FT and FPN levels decrease. Moreover, STEAP (54) and DMT1 are upregulated (57). Ultimately, these changes lead to an increase in intracellular iron (58).

In cancer and inflammation, hepcidin secretion from the liver and peripheral tissues increases, leading to decreased FPN expression, which limits iron circulation and contributes to anemia. Cancer cells exhibit an “iron-seeking” phenotype by upregulating iron-related proteins (TFR1, STEAP, DMT1, hepcidin, SLC39A14) and downregulating FPN, thereby promoting iron uptake and accumulation (59). Studies have shown that exogenous iron supplementation, in the form of ammonium ferric citrate at submillimolar doses, induces ROS and lipid peroxidation in mitochondria (60). This also suggests that intracellular iron accumulation increases cellular susceptibility to ferroptosis.

#### 2.2.2 Noncanonical ferroptotic pathway

##### 2.2.2.1 p53 signaling in ferroptosis

p53 is a classic tumor suppressor that plays an important role in cell cycle regulation, apoptosis, metabolic regulation and maintenance of genomic stability (61). p53 directly inhibits the expression of SLC7A11, limiting the uptake of L-cysteine, thereby reducing the synthesis of GSH and reducing the activity of GPX4 (62).In addition, SAT1 and GLS2 are transcriptional targets of p53. Studies have shown that p53 enhances ferroptosis by upregulating the expression of SAT1 and GLS2 (63). p53 can also enhance iron uptake and increase the accumulation of the LIP by upregulating the expression of TFR1 (64). This effect makes the iron-dependent Fenton reaction more likely to occur, thus generating more hydroxyl radicals (•OH) and inducing lipid peroxidation. In addition, p53 promotes lipid remodeling of intracellular PUFAs by regulating the expression of ACSL4, thereby exacerbating lipid peroxidation (65).

##### 2.2.2.2 Ferritinophagy and autophagy-dependent ferroptosis

Ferritinophagy is a form of selective autophagy that targets ferritin for degradation, releasing stored iron and increasing the level of the LIP (66).During ferritinophagy, the ferritin receptor nuclear receptor coactivator 4(NCOA4) binds to ferritin and transports it to the autophagosome for degradation, releasing the stored iron. Studies have shown that inhibiting NCOA4 or blocking ferritinophagy can significantly reduce ferroptosis (67).

In some cases, autophagy can enhance the sensitivity of cells to ferroptosis. Autophagy-related genes (ATG5, ATG7) significantly affect the occurrence of ferroptosis by promoting mitochondrial autophagy and regulating the level of lipid peroxidation (68, 69). Additionally, studies have shown that overactivation of autophagy may accelerate ferritinophagy and the release of free iron, promoting the Fenton reaction and lipid peroxidation chain reaction, which in turn exacerbates ferroptosis (70).

##### 2.2.2.3 Endoplasmic Reticulum Stress and ferroptosis

Endoplasmic Reticulum Stress (ERS) is a cellular stress response triggered by protein misfolding or calcium imbalance in the ER. Studies have shown that ERS plays a crucial role in cell death by regulating ferroptosis-related molecules and signaling pathways (71).

**Figure 1:**
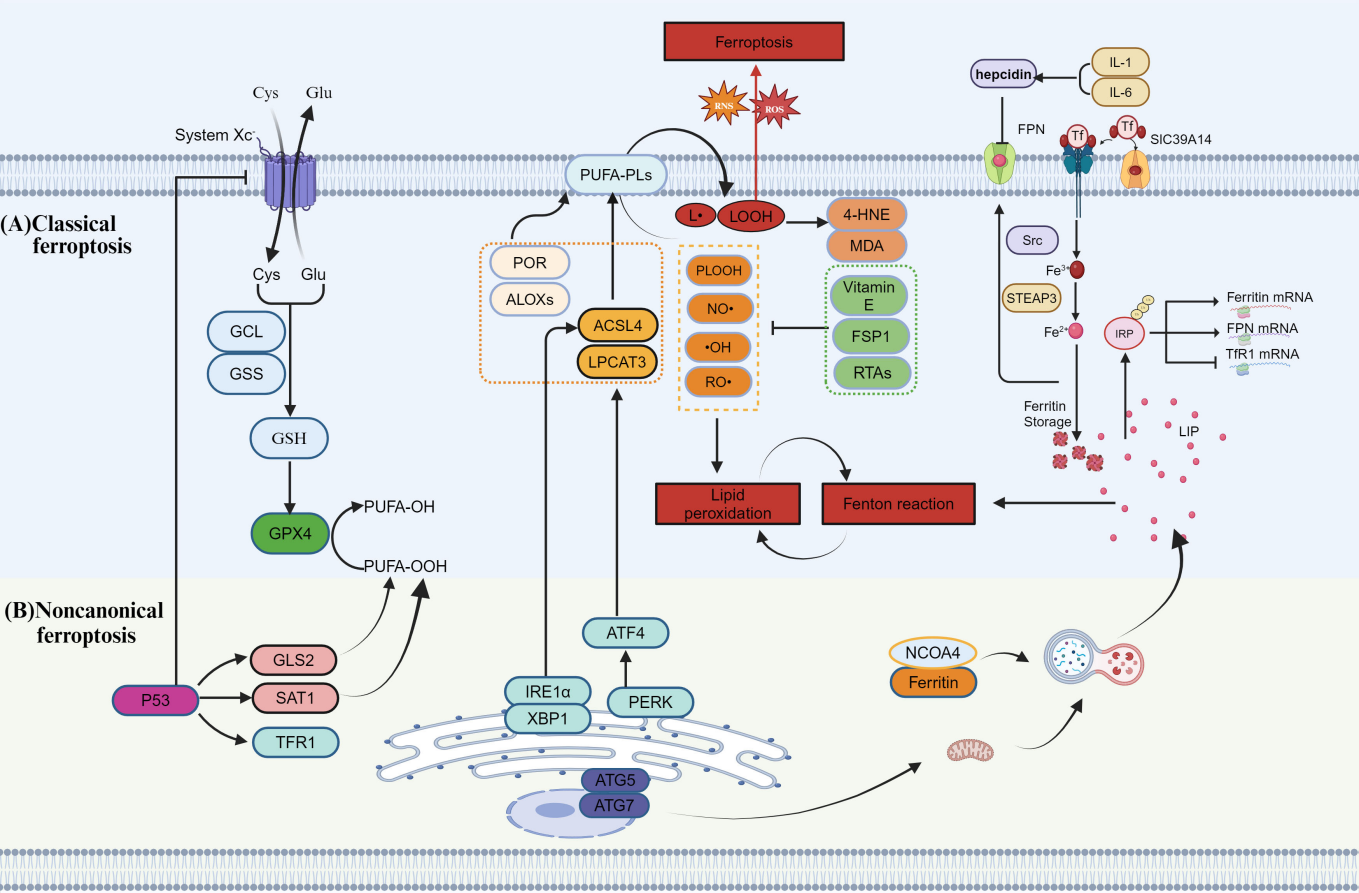
Regulation of ferroptotic signaling **(A)** Classical Ferroptosis Pathway: This pathway primarily involves the synthesis of GSH and the reduction of lipid hydroperoxides (LOOH) by GPX4, which prevents ferroptosis. Cells take up cysteine (Cys) through the System Xc- to support GSH synthesis. GSH then inhibits the propagation of lipid peroxidation through GPX4, avoiding ferroptotic cell death. Polyunsaturated fatty acids (PUFAs) and phospholipids (PLs) play crucial roles in this process, with lipoxygenases (ALOXs) and other enzymes like POR, ACSL4, and LPCAT3 driving lipid peroxidation. **(B)** Noncanonical Ferroptosis Pathway: This pathway involves different molecular mechanisms, including the regulation of p53, GLS2, and SAT1, and affects ferroptosis through the expression of transferrin receptor 1 (TFR1) and ferritin. Endoplasmic reticulum stress (ERS) and autophagy also play significant roles in ferroptosis, with key players like ATG5 and ATG7 regulating the autophagic process.

Studies have found that the ER stress-induced IRE1α-XBP1 signaling pathway regulates the expression of ACSL4, promotes the phospholipidation of PUFAs, enhances Lipid Peroxidation(LPO), and aggravates ferroptosis (72, 73). ER stress promotes ferroptosis by activating the PERK pathway, upregulating the expression of ATF4, and further enhancing the expression of ferroptosis-driving factors, ACSL4 and LPCAT3 (74, 75). Overactivation of ATF4 may also induce the downregulation of SLC7A11, reduce intracellular GSH levels, and further aggravate ferroptosis (76). Regulation of ferroptotic signaling is shown in **Figure 1**.

## 3 Role of ferroptosis in OC

### 3.1 Ferroptosis in the onset and metastasis of OC

**Figure 2:**
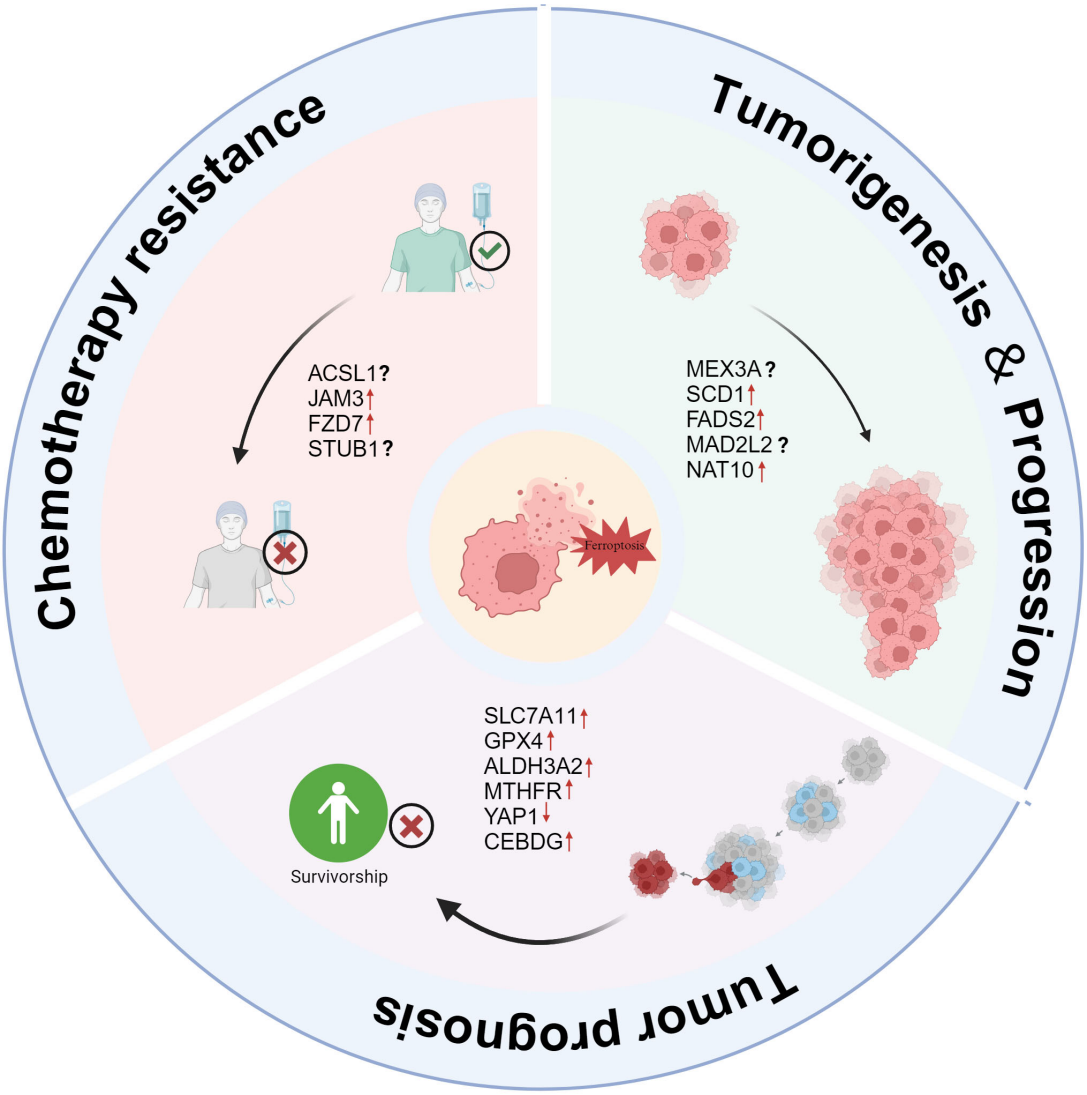
The role of ferroptosis in OC tumorigenesis, progression, and chemotherapy resistance. Tumorigenesis & Progression: Several factors, including MEX3A, SCD1, FADS2, MAD2L2, and NAT10, contribute to OC development and progression by suppressing ferroptosis, promoting tumor growth. Chemotherapy Resistance: ACSL1, JAM3, FZD7, and STUB1 are linked to resistance against chemotherapeutic agents, potentially reducing ferroptosis susceptibility and enabling tumor survival. Tumor Prognosis: High expression of SLC7A11, GPX4, ALDH3A2, MTHFR, YAP1, and CEBPG correlates with poor prognosis by inhibiting ferroptosis, whereas ferroptosis induction could improve patient outcomes.

OC is widely considered to be a “hidden killer” of women. Due to its high mortality rate, it remains one of the leading causes of cancer-related deaths in women worldwide. Recent studies have found that ferroptosis, a special type of programmed cell death, plays a significant role in the occurrence, development and metastasis of OC (77, 78). Ferroptosis causes cell death by inducing intracellular oxidative stress and destroying lipid peroxides on the cell membrane. Acording to **Figure 2**, this mechanism plays a crucial role in the proliferation, drug resistance and metastasis of OC cells (79, 80).

In a study exploring the different cellular origins and pathogenesis of endometriosis-related OC (CCOC and ENOC), Ian Beddows et al. found (81) that CCOC cells enhance the gene expression of cysteine and GSH synthesis pathways and downregulate iron transporters. Battaglia, A.M. et al. also found that (82) iron metabolism plays a key role in the sphere-forming ability of OC cells. Under non-adhesive culture conditions, increased iron promoted the growth and maintenance of OC stem-like cells, while iron chelators significantly inhibited this process.

OC cells promote survival by inhibiting ferroptosis, a mechanism that not only facilitates the early initiation of tumors but also enables them to remain invasive during progression. For example, MEX3A promotes the proliferation of OCCC cells and tumor progression by regulating the stability of wild-type p53 and inhibiting ferroptosis (83). In addition, abnormal upregulation of SCD1 and FADS2 enhances lipid metabolism activity and tumor invasiveness in ascites-derived OC cells by inhibiting ferroptosis (84). Furthermore, studies have shown that OC metastasis-derived cells exhibit higher ferroptosis sensitivity and increased PUFA content, suggesting a close link between ferroptosis and the potential for OC metastasis (85). The relationship between ferroptosis susceptibility and poor prognosis in OC has also been further verified. Additionally, other molecules, such as MAD2L2 and NAT10, also promote OC progression by inhibiting the ferroptosis pathway. As a key regulatory factor, MAD2L2 further accelerates tumor progression by suppressing ferroptosis (86). Upregulation of NAT10 promotes ovarian tumor development by enhancing fatty acid metabolism and inhibiting ferroptosis (87). These findings suggest that ferroptosis plays a critical role in the onset and metastasis of OC. Therefore, regulating ferroptosis and its associated signaling pathways may offer new insights and strategies for the clinical treatment of OC. In summary, ferroptosis and its related regulatory molecules could become promising targets for future OC therapies.

### 3.2 Ovarian cancer tumor microenvironment and ferroptosis

The tumor microenvironment (TME) is a dynamic, complex system that significantly influences cancer progression and therapeutic response. Recent studies have highlighted ferroptosis, a novel form of programmed cell death, as being regulated by various components of the TME, particularly through interactions among immune cells, stromal cells, and the extracellular matrix (88–90). These intricate interactions not only impact the effectiveness of ferroptosis inducers but also shape the tumor’s overall biological characteristics. For instance, Wei C et al.’s (91)immune and ferroptosis-related risk scoring model underscores the pivotal role of ferroptosis within the TME. Similarly, the prognostic risk model developed by Gao J et al. (92), which focuses on ferroptosis-related long noncoding RNAs (lncRNAs), further demonstrates how these lncRNAs regulate the TME in ovarian cancer.

A key interaction in the TME involves the regulation of immune cells, particularly macrophages. Ji H-Z et al. (93) discovered that in ovarian cancer, tumor-associated macrophages secrete CXCL8, activate CXCR2, and upregulate the expression of SLC7A11 and GPX4 in endothelial cells via the NF-κB signaling pathway, thereby protecting endothelial cells from ferroptosis. This mechanism not only explains ferroptosis resistance within the TME but also underscores the crucial role of ferroptosis in tumor progression. Additionally, Wang Y et al. (94) identified that growth factors such as FGF2, 3, 8, 17, 18, 19, and 23 are closely linked to the ferroptosis signaling pathway in ovarian cancer. Their study found that FGF co-expresses with ferroptosis-related genes like GPX4, FTH1, and HMOX1, and that the level of FGF expression is negatively correlated with immune cell infiltration. This suggests that FGF influences ovarian cancer progression by modulating iron metabolism and immune responses. Li Z et al. (95) also pointed out that in the fatty acid-rich TME of ovarian cancer, the upregulation of SLC27A4 enhances tumor cell resistance to ferroptosis by promoting the selective uptake of MUFA, thereby inhibiting lipid peroxidation. Moreover, Atiya HI et al. (96)found that endometriosis-derived mesenchymal stem cells (enMSC) contribute to ovarian clear cell carcinoma (OCCC) growth through iron regulation. Specifically, when CD10-negative enMSC were co-cultured with OCCC cells, significant changes were observed in iron transport and export, highlighting the crucial role of iron homeostasis in the TME for ovarian cancer progression.

### 3.3 Chemoresistance and ferroptosis in OC

As is shown in **Figure 2**, ferroptosis plays a crucial role in the chemoresistance of OC (97, 98). Various molecular mechanisms work together to suppress ferroptosis, thereby enhancing cancer cell resistance to chemotherapy. However, research indicates that targeting these resistance-related pathways can restore ferroptosis sensitivity and improve chemotherapy efficacy.

ACSL1 enhances the stability and activity of FSP1 by promoting its N-myristoylation, thereby inhibiting lipid peroxidation and ferroptosis. This mechanism contributes to significant resistance to platinum-based chemotherapy in OC cells. Notably, Zhang et al. found that inhibiting ACSL1 or targeting the N-myristoylation process of FSP1 can restore ferroptosis sensitivity and significantly improve the efficacy of platinum-based drugs (99). Meanwhile, JAM3, a transmembrane protein from the junctional adhesion molecule family, plays a crucial role in cell adhesion and signal transduction, particularly in cell migration and tumor metastasis (100, 101). Studies have shown that high JAM3 expression is closely associated with cisplatin resistance and poor prognosis in OC patients. Specifically, JAM3 enhances the resistance of highly adhesive OC cells to ferroptosis by activating the NRF2/FSP1 pathway and inhibiting lipid peroxidation. Targeting the JAM3-NRF2/FSP1 pathway presents a promising therapeutic strategy to overcome drug resistance and increase ferroptosis sensitivity (102).Additionally, Frizzled-7 (FZD7) is a key marker of platinum-resistant OC cells (103, 104). Wang et al. (105) found that OC cells maintain redox balance and resist oxidative damage by activating the FZD7–β-catenin–Tp63–GPX4 signaling pathway, further promoting chemotherapy resistance. Therefore, targeting FZD7 and its downstream pathways may be an effective strategy for improving chemotherapy sensitivity. Furthermore, as a ubiquitin ligase, STUB1 plays a crucial role in regulating ferroptosis in OC cells and enhancing sensitivity to the chemotherapy drug paclitaxel. STUB1 promotes ferroptosis by mediating the ubiquitination and degradation of HOXB3, thereby inhibiting PARK7 expression (106). Understanding this mechanism provides a potential new approach for overcoming paclitaxel resistance in OC and lays the groundwork for future targeted therapy research.

In summary, the inhibition of ferroptosis is one of the important mechanisms of OC resistance. With the in-depth study of these molecular mechanisms, more and more evidence shows that targeting these key regulatory factors can effectively restore the sensitivity of OC cells to ferroptosis and improve the therapeutic effect of chemotherapy drugs. In the future, intervention strategies targeting these molecular mechanisms may become an effective way to break through the bottleneck of OC resistance and bring more clinical benefits to patients with resistant OC.

### 3.4 OC prognosis and ferroptosis

Ferroptosis is closely related to the metabolic regulation of tumor cells, drug resistance and the prognosis of patients (**Figure 2**). The importance of ferroptosis-related molecules in the prognosis of OC has been demonstrated by an increasing number of studies. Wu, X et al. found that high levels of SLC7A11 and GPX4 co-expression are important predictors of platinum resistance and poor prognosis in patients with epithelial OC. According to mechanistic research, SLC7A11 and GPX4 stop ferroptosis by boosting cells’ antioxidant potential and preventing lipid peroxidation (107). ALDH3A2 is an enzyme involved in lipid metabolism that catalyzes the oxidation of long-chain fatty aldehydes to fatty acids, thereby regulating cellular lipid homeostasis and antioxidant capacity. Studies have shown that ALDH3A2 affects the sensitivity of tumor cells to ferroptosis by regulating lipid metabolism, GSH metabolism, phospholipid metabolism, and aldehyde metabolism pathways (108). Using TCGA and GTEx data, Dong, H. et al. also discovered that a poor prognosis for patients with OC was linked to high expression of ALDH3A2 (109). In addition to being closely linked to intracellular methylation processes, MTHFR is a crucial metabolic enzyme involved in the metabolism of folate and homocysteine (110). According to studies, women with OC who have high MTHFR expression had a far worse prognosis (111). Wang, X. et al. also discovered that MTHFR knockdown can greatly increase the anti-tumor effect and boost the lethal effect of ferroptosis inducers (such Erastin and RSL3) (112). In addition to metabolic regulation, transcription factors also play an important role in ferroptosis-related prognostic mechanisms. According to Furutake, Y. et al., YAP1 activation greatly increased the sensitivity of OCCC cells to ferroptosis inducers (such Erastin) via boosting lipid peroxidation, and low nuclear YAP1 expression was strongly linked to a poor prognosis in OCCC (113). This discovery suggests that YAP1 may play a part in controlling ferroptosis during the treatment of OC. Zhang et al. found that CEBPG expression is significantly higher in OC tissue than in benign ovarian tissue and is associated with poor prognosis in OC patients. Further studies revealed that CEBPG inhibits ferroptosis by regulating SLC7A11 transcription, thereby promoting the proliferation, migration, and invasion of OC cells (114).

These ferroptosis-related molecules not only reveal the key biological mechanisms for the prognosis of OC patients, but also provide new directions for precision treatment strategies targeting ferroptosis.

### 3.5 Molecular mechanisms and regulatory networks of ferroptosis in OC

#### 3.5.1 The role of miRNA, lncRNA, and circRNA in ferroptosis of OC

**Figure 3:**
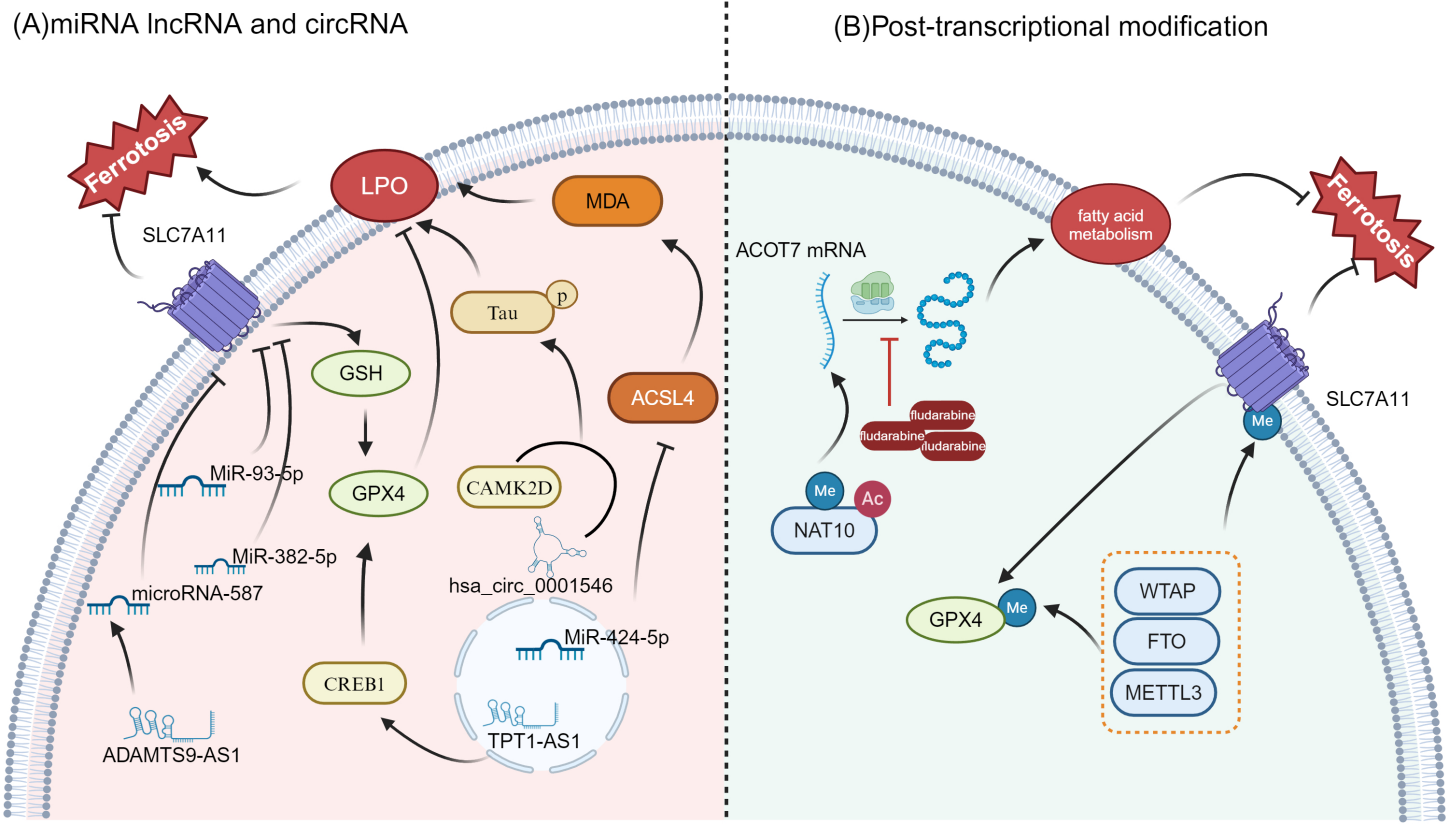
Regulation of ferroptosis by miRNAs, lncRNAs, circRNAs, and post-transcriptional modifications **(A)** miRNA, lncRNA, and circRNA regulation in ferroptosis: Key miRNAs like MiR-93-5p, MiR-382-5p, and MiR-424-5p influence ferroptosis by modulating critical genes such as SLC7A11, GPX4, and ACSL4. hsa_circ_0001546 and TPT1-AS1 also contribute to lipid peroxidation (LPO) and ferroptosis sensitivity. Additionally, CREB1 plays a role in ferroptosis regulation through lipid metabolism and by impacting ACSL4 activity **(B)** Post-transcriptional Modifications: Modifications such as methylation (Me) and acetylation (Ac) are involved in regulating the expression and function of key proteins like SLC7A11 and GPX4, both of which are essential for ferroptosis regulation. The enzyme NAT10 influences fatty acid metabolism and ferroptosis by modifying mRNA stability, particularly for genes like ACOT7. In addition, methyltransferases such as METTL3, FTO, and WTAP are critical in the regulation of GPX4 and SLC7A11, further modulating the ferroptotic process.

As is shown in **Figure 3**, the occurrence of ferroptosis in OC cells is closely related to the expression of multiple RNA molecules, among which miRNA, lncRNA and circRNA play an important role as regulatory factors in the sensitivity and tolerance of ferroptosis. These RNA molecules influence the ferroptosis process through distinct mechanisms, significantly impacting the growth, migration, invasion, and prognosis of OC.

MiR-93-5p is a small noncoding RNA belonging to the microRNA (miRNA) family. Li, C et al. reported that miR-93-5p down-regulated SLC7A11, reduced GSH levels, increased the accumulation of ROS and lipid peroxides, and ultimately induced ferroptosis (115). In addition, Sun et al. showed that miR-382-5p can promote ferroptosis by inhibiting SLC7A11 and may become a potential target for the future treatment of OC (116). Another microRNA, miR-424-5p, can reduce the accumulation of lipid peroxidation end products (such as MDA) and reduce ferroptosis-related cell death by directly targeting ACSL4. In contrast, inhibition of miR-424-5p can enhance the sensitivity of OC cells to ferroptosis (117).

Through multi-omics analysis, Wang, K. et al. revealed the significant role of ferroptosis-related long noncoding RNAs (lncRNAs) in OC. Eight ferroptosis-related lncRNAs were screened (such as RP11-443B7.3, TRAM2-AS1, FAM13A-AS1, AC107959.3, AC068870.2, ACBD3-AS1, LINC01857, and AL031186.1), and were found to be closely associated with the prognosis of OC patients. A risk scoring model based on these lncRNAs was constructed to assess the potential benefits of immunotherapy (118). In addition, TPT1-AS1, a long noncoding RNA, promotes the transcription of GPX4 by upregulating the transcription factor CREB1, thereby inhibiting ferroptosis and enhancing the proliferation, migration, and invasion of OC cells (119). In epithelial OC (EOC), ADAMTS9-AS1 upregulation indirectly promotes SLC7A11 expression and inhibits ferroptosis by suppressing microRNA-587. Silencing ADAMTS9-AS1, on the other hand, increases microRNA-587 levels, leading to reduced SLC7A11 expression, which in turn promotes ferroptosis and inhibits the proliferation and migration of OC cells (120).

In terms of circular RNA regulation, Chai, B. et al. reported that hsa_circ_0001546, functioning as a molecular chaperone for the 14-3–3 protein, interacts with CAMK2D to promote the abnormal phosphorylation of Tau protein at Ser324, thereby driving lipid peroxidation-dependent ferroptosis. This mechanism effectively inhibits the proliferation and metastasis of OC cells, offering a potential new strategy for targeted therapy (121).

#### 3.5.2 Post-transcriptional modifications and ferroptosis

Recent studies have increasingly highlighted the crucial role of post-transcriptional modifications in regulating ferroptosis, as well as in tumorigenesis and cancer progression (**Figure 3**) (122–124). A study found that NAT10 enhances the stability and translation of ACOT7 mRNA through m6A-driven translational regulation and ac4C RNA modification, thereby remodeling fatty acid metabolism, inhibiting ferroptosis and promoting the occurrence and progression of OC. More importantly, the study pointed out that the small molecule inhibitor fludarabine can target NAT10 and inhibit the acetylation modification of ACOT7 mRNA, inhibiting the acetylation modification of ACOT7 mRNA and effectively preventing tumor development. This offers a promising new strategy for treating OC (87).

Other studies have also highlighted the critical role of post-transcriptional modifications in regulating ferroptosis. For instance, research has shown that m6A modification influences ferroptosis sensitivity by modulating the expression of key genes, such as GPX4 and SLC7A11 (125). m6A-related enzymes such as METTL3, WTAP, and FTO affect mRNA stability and translation efficiency by regulating RNA methylation modification, thereby participating in the regulation of lipid peroxidation and cell ferroptosis (126, 127). These findings further suggest that post-transcriptional modifications play an important role in regulating ferroptosis in OC cells, offering new insights for potential therapeutic strategies.

#### 3.5.3 Ferroptosis inhibitors and promoters

##### 3.5.3.1 Ferroptosis inhibitors

NRF2 is a transcription factor that helps maintain cellular redox balance by regulating antioxidant gene expression. It plays a crucial role in responding to oxidative stress, inflammation, and cellular metabolism (128). Studies show that NRF2 impacts iron homeostasis in OC cells by regulating the expression of HERC2 and VAMP8. Specifically, NRF2 upregulates HERC2 expression, promoting ferritin degradation and increasing free iron levels. Conversely, NRF2 downregulates VAMP8 expression, inhibiting ferritin autophagy, and thereby modulating ferroptosis sensitivity (129). Additionally, **SGK1** (serum and glucocorticoid-induced kinase 1) inhibits ferroptosis in OC through both NRF2-dependent and -independent pathways. Sang et al. reported that SGK1 enhances antioxidant defense and reduces lipid peroxide production by activating the NRF2 pathway. Furthermore, SGK1 directly regulates intracellular iron and lipid metabolism through NRF2-independent pathways, further inhibiting ferroptosis (130).

SLC2A12 and SLC27A4 also contribute to cellular metabolism. Under hypoxic conditions, HIF-1A binds to HIF-1B and upregulates SLC2A12 expression by binding to the hypoxia response element (HRE) in the SLC2A12 promoter. This regulation enhances GSH metabolism, promotes GPX4 expression, and inhibits lipid peroxide formation, thereby preventing ferroptosis (131). In contrast, SLC27A4 inhibits lipid peroxide production and ferroptosis in OC by promoting monounsaturated fatty acids (MUFA) and phospholipid remodeling (123). PTRF/Cavin-1 is a protein involved in cell membrane structure and signal transduction. It inhibits ferroptosis by maintaining cell membrane integrity and lipid homeostasis. Xiang et al. identified PTRF/Cavin-1 as a key regulator in OC, preventing ferroptosis. Knockdown of PTRF/Cavin-1 increased OC cells’ sensitivity to ferroptosis inducers, such as Erastin and RSL3 (132).

**Figure 4:**
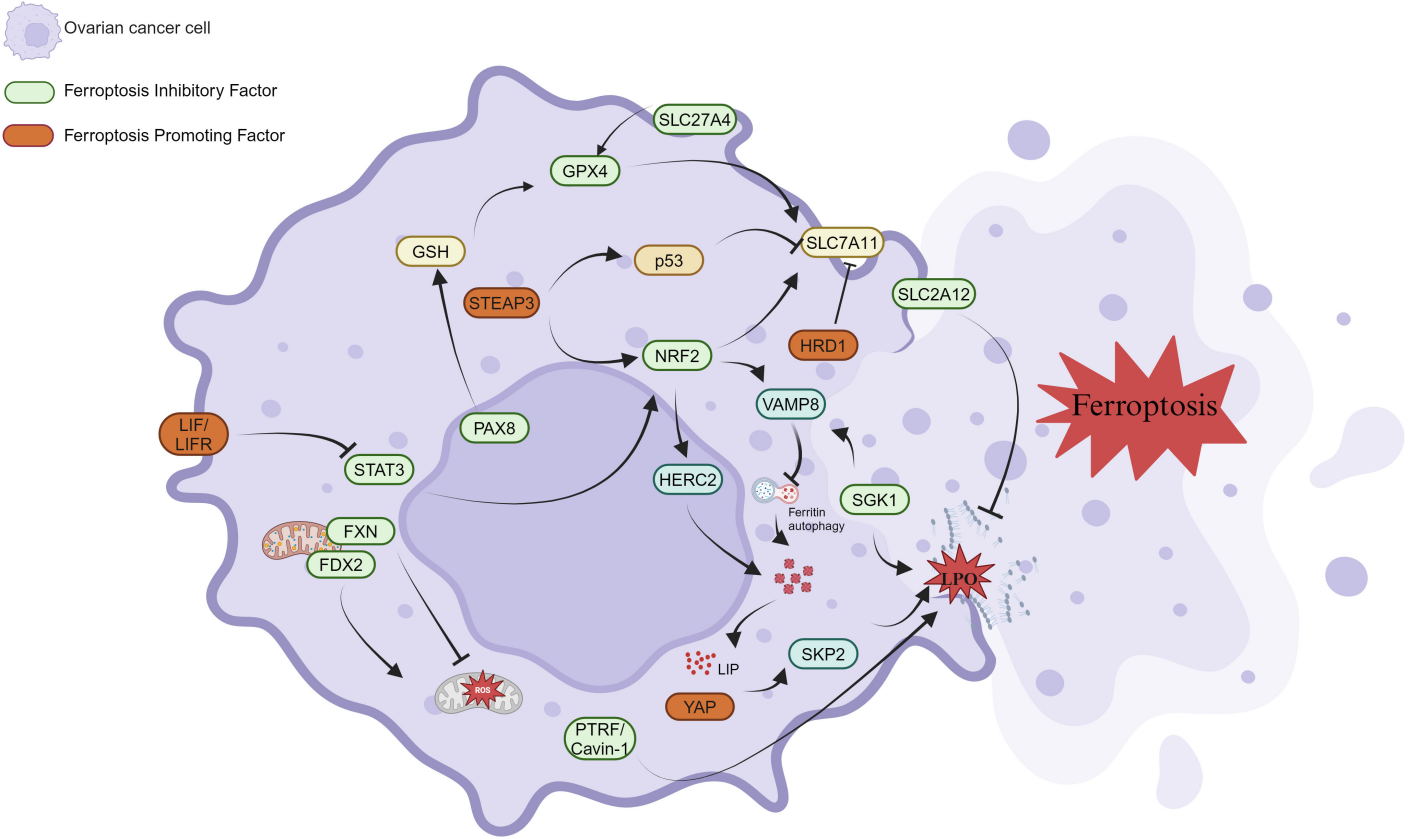
Ferroptosis regulatory network in OC cells. Ferroptosis-promoting factors (highlighted in red) include LIF/LIFR, STAT3, HRD1, and SKP2, which are involved in regulating iron metabolism, oxidative stress, and lipid peroxidation (LPO), all of which contribute to ferroptosis. SLC7A11, SLC2A12, and STEAP3 play important roles in iron uptake and GSH synthesis, while VAMP8 and ferritin autophagy regulate intracellular iron storage. Ferroptosis inhibitory factors (highlighted in green) such as GPX4, NRF2, PAX8, and p53 help prevent ferroptosis by maintaining cellular redox balance and suppressing lipid peroxidation. FXN, FDX2, and PTRF/Cavin-1 contribute to iron homeostasis and mitochondrial function.

PAX8, a transcription factor, enhances the antioxidant capacity of OC cells by upregulating genes related to GSH synthesis, such as GCLC and GSS, thereby inhibiting ferroptosis (133). FXN, a mitochondrial iron-sulfur cluster assembly protein, regulates the degradation of mitochondrial antioxidant protein PRDX3 and inhibits ferroptosis in OC stem cells. FXN deficiency increases ROS accumulation, disrupts the intracellular redox balance, and induces ferroptosis, impairing the survival and self-renewal of tumor stem cells (134). Moreover, Miyahara et al. found that FDX2 prevents ferroptosis, cell senescence, and apoptosis in OC cells by maintaining mitochondrial iron-sulfur cluster homeostasis. Knockdown of FDX2 causes ROS accumulation and increased lipid peroxidation, significantly heightening the sensitivity of OC cells to ferroptosis inducers. *In vivo* experiments also demonstrated that the loss of FDX2 inhibited tumor growth (135). Finally, SQLE, the rate-limiting enzyme in the cholesterol biosynthesis pathway, is significantly overexpressed in OC (136, 137). Zhang et al. discovered that SQLE inhibits lipid peroxide generation and ROS accumulation by regulating cholesterol and lipid metabolism, reducing ferroptosis occurrence. Knockdown of SQLE significantly increased the sensitivity of OC cells to ferroptosis inducers, such as GPX4 inhibitors, and markedly inhibited tumor growth in *in vivo* experiments (138). The ferroptosis inhibitors is shown in **Figure 4**.

##### 3.5.3.2 Ferroptosis promoting factors

STEAP3, a key regulator of iron metabolism, has been shown by Han, Y. et al. to inhibit the expression of SLC7A11 through activation of the p53 signaling pathway. This reduces GSH levels, increases lipid peroxidation and ROS accumulation, ultimately inducing ferroptosis and inhibiting OC progression (139). The LIF/LIFR autocrine loop enhances OC cells’ sensitivity to ferroptosis by suppressing the antioxidant defense system downstream of the STAT3/NRF2/SLC7A11-GPX4 axis. Ebrahimi, B. et al. demonstrated that the LIFR inhibitor EC359 not only inhibited OC cell growth and stem cell properties, but also induced lipid peroxidation and cell death by downregulating anti-ferroptosis genes, ultimately slowing tumor progression in both *in vivo* and *in vitro* models (140). HRD1 inhibits tumor proliferation and metastasis by promoting the ubiquitination and degradation of SLC7A11. Wang, Y. et al. found that elevated HRD1 expression increases ROS and lipid peroxidation levels, triggers ferroptosis, and limits tumor growth (141). In addition, Yang, W.-H. et al. reported that the Hippo pathway effector YAP promotes ferroptosis by regulating the E3 ubiquitin ligase SKP2. Knockdown of YAP significantly decreased the sensitivity to lipid peroxidation and ferroptosis, while genetic or chemical inhibition of SKP2 protected cancer cells from ferroptosis. This discovery reveals the critical regulatory roles of YAP and SKP2 in ferroptosis and offers new therapeutic avenues for targeting YAP or SKP2 in cancer treatment (142). The ferroptosis promoters is shown in **Figure 4**.

## 4 Preclinical strategies for ferroptosis-based OC therapy

**Figure 5:**
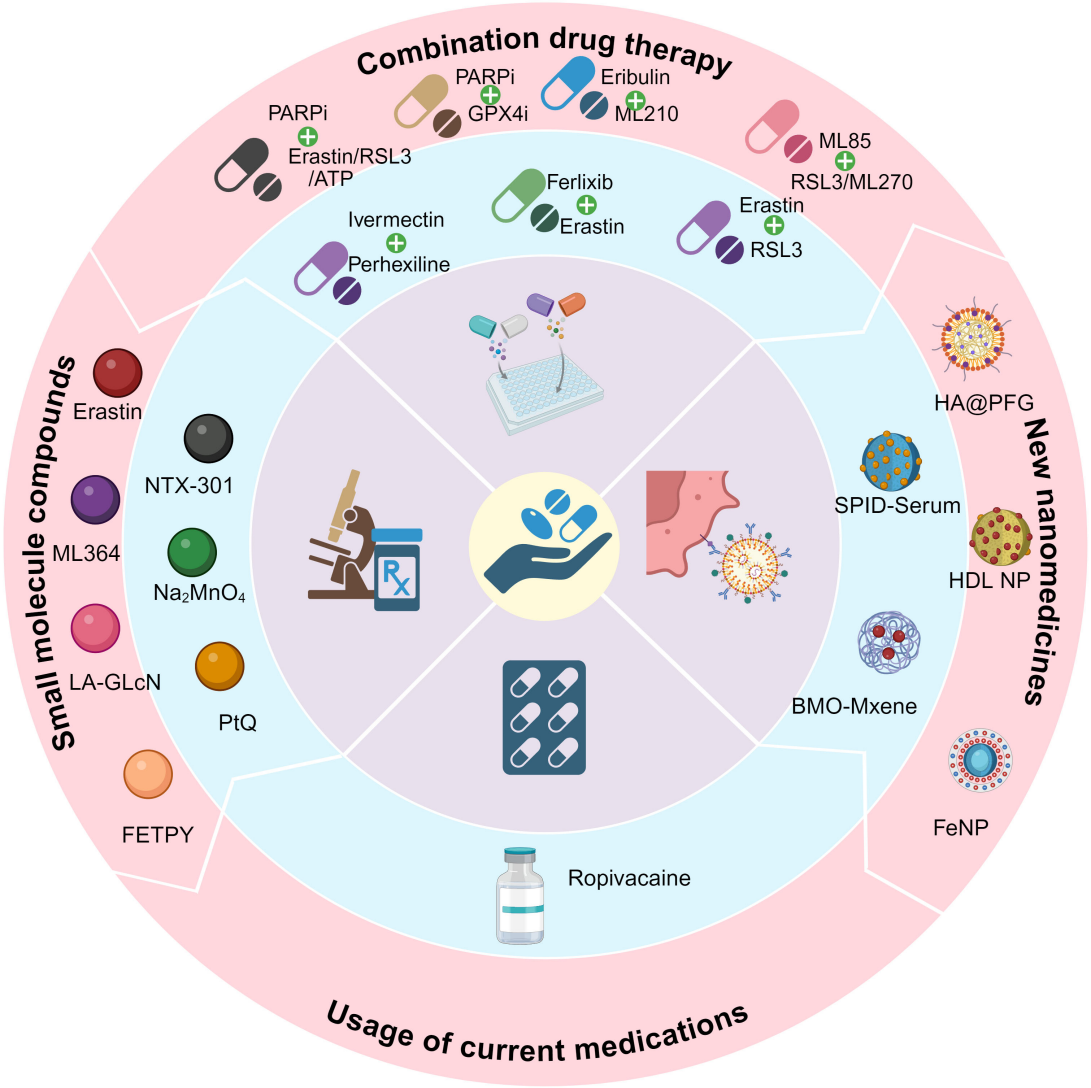
Schematic diagram of the classification of therapies for OCs.

**Figure 6:**
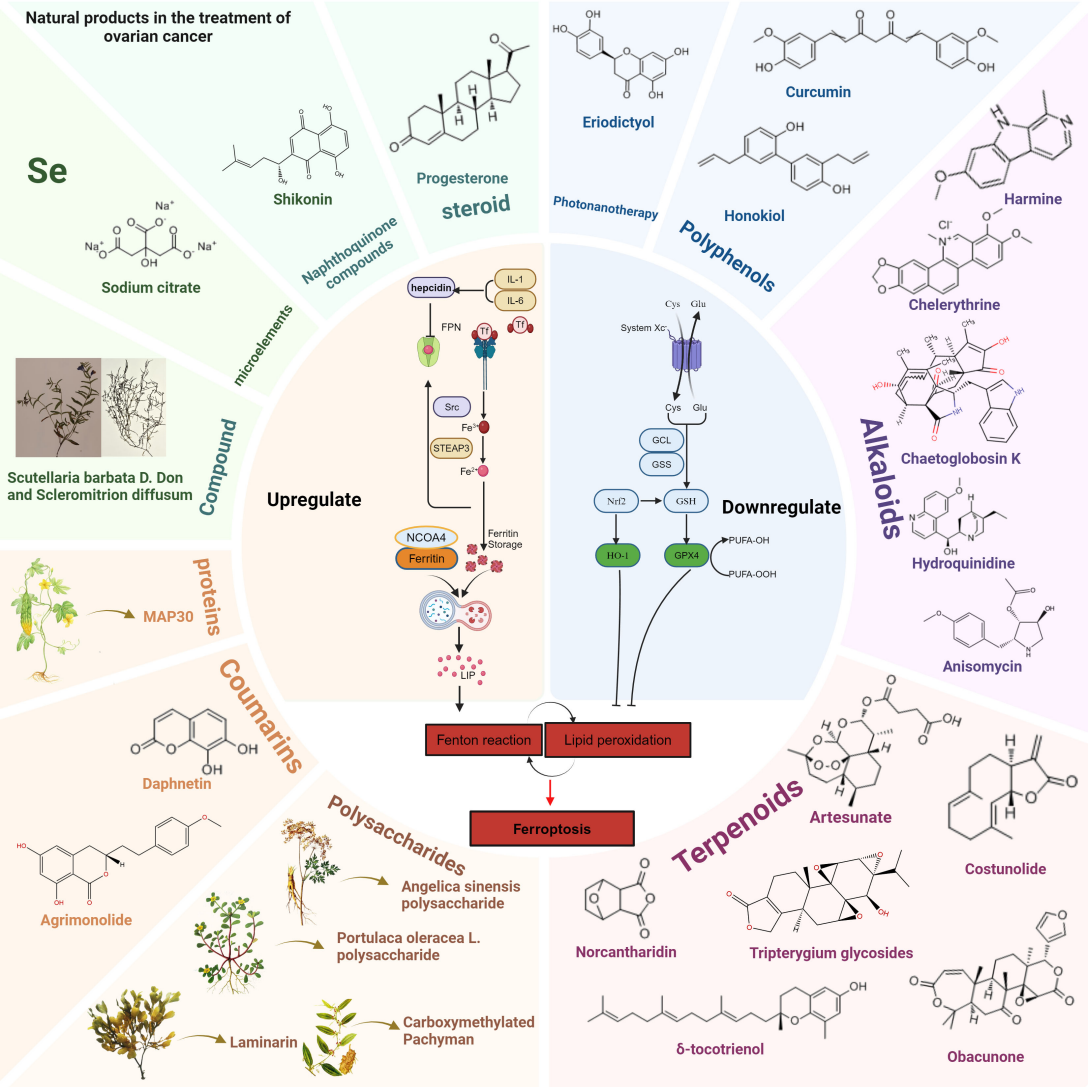
Natural products modulate ferroptosis in OC by upregulating or downregulating key molecular pathways. Natural compounds are categorized into different groups, including polyphenols, alkaloids, terpenoids, steroids, naphthoquinones, coumarins, polysaccharides, proteins, trace elements (Se), and traditional Chinese medicine (Scutellaria barbata D. Don and Scleromitrion diffusum). Left Panel: Compounds that upregulate ferroptosis, such as sodium citrate, shikonin, and progesterone, promote lipid peroxidation and Fenton reactions, increasing free iron and oxidative stress. Key proteins such as NCOA4 mediate ferritinophagy, leading to intracellular iron accumulation. Right Panel: Compounds that downregulate ferroptosis, such as curcumin, honokiol, and eriodictyol, act by enhancing antioxidant pathways. These include upregulation of GPX4, HO-1, and GSH metabolism, reducing lipid peroxidation and preventing ferroptotic cell death.

**Figures 5** and **6** show the preclinical strategies for ferroptosis-based OC treatment.

### 4.1 Combination drug therapy for OC

In recent years, OC treatment has increasingly focused on combination therapies, particularly the use of ferroptosis inducers alongside chemotherapy drugs. This approach has shown promise in enhancing treatment efficacy and overcoming drug resistance. For example, Battaglia et al. reported that Ferlixit can mitigate Erastin resistance by increasing intracellular iron levels, inducing mitochondrial dysfunction, and activating lipid peroxidation. Thus, the combination of Erastin and Ferlixit can synergistically enhance ferroptosis induction (82). The combination of Erastin and RSL3 significantly enhances cytotoxicity, particularly in multidrug-resistant and Ras-mutated OC cells, demonstrating strong therapeutic potential (143). Meanwhile, NRF2 and GPX4 play key roles in the antioxidant defense system of OC cells. Studies have shown that co-administering the NRF2 inhibitor ML85 with the GPX4 inhibitors RSL3 and ML210 induces lipid peroxidation and ROS accumulation, triggering both ferroptosis and apoptosis. This combination effectively inhibits OC cell growth and metastasis. Further animal studies confirm its efficacy in reducing abdominal implanted tumors, offering a promising strategy for OC treatment (144).Additionally, Eribulin, a non-taxane microtubule inhibitor widely used for advanced breast and OC, has shown effectiveness in patients resistant to other treatments. Research indicates that Eribulin induces ferroptosis in ovarian clear cell carcinoma (OCCC) cells by enhancing ROS generation, increasing intracellular iron levels, promoting lipid peroxidation, and inhibiting the NRF2-HO-1 antioxidant pathway. Furthermore, Eribulin exacerbates ferroptosis by disrupting mitochondrial function and reducing DHODH protein expression. Its combination with the GPX4 inhibitor ML210 further enhances its anti-tumor effects, presenting a potential therapeutic target for OCCC (145).

Targeting DNA repair pathways, the PARP inhibitor Olaparib has also shown potential in regulating ferroptosis. Hong T. et al. found that Olaparib suppresses SLC7A11 expression and reduces GSH synthesis, thereby promoting lipid peroxidation and ferroptosis in OC. Further studies indicate that combining PARP inhibitors with ferroptosis inducers significantly enhances treatment sensitivity in BRCA wild-type OC (146). Additionally, the combination of Olaparib and arsenic trioxide (ATO) has been shown to enhance chemotherapy sensitivity in platinum-resistant OC by promoting both ferroptosis and apoptosis. Specifically, ATO strengthens Olaparib’s anti-cancer effects by activating the AMPK pathway and inhibiting SCD1, offering a promising combination therapy strategy to overcome platinum resistance (147). BRCA1 also enhances OC cell sensitivity to ferroptosis by promoting GPX4 degradation through K6-linked polyubiquitination. Conversely, BRCA1 deficiency leads to increased GPX4 levels, inhibiting ferroptosis and promoting tumor growth. In BRCA1-deficient OC cells, PARP inhibitors (PARPi) can induce ferroptosis, and their combination with GPX4 inhibitors exhibits significant synergistic anti-tumor effects. This approach presents a promising strategy for optimizing the treatment of BRCA1-deficient cancers (148). Additionally, in the context of drug resistance, HSP27 and fatty acid oxidation (FAO) reduce ROS levels induced by cisplatin, thereby inhibiting its cytotoxic effects in platinum-resistant OC. Studies have shown that combining ivermectin and perhexiline to inhibit HSP27 and FAO significantly enhances cisplatin’s effectiveness against drug-resistant OC cells. This finding suggests that targeting HSP27 and FAO could offer a promising strategy for overcoming drug resistance in OC treatment (149).

In summary, combining ferroptosis inducers with chemotherapy or targeted therapies can enhance the sensitivity of OC cells to treatment while overcoming drug resistance, offering new options for clinical management. However, further research is needed to evaluate the long-term effects, safety, and impact of these combination strategies on the tumor microenvironment. Such studies will help develop more precise and effective treatments for OC patients.

### 4.2 Development of new nanomedicines

The creation of medications based on nanomaterials has created new avenues for the treatment of OC in recent years. When paired with the ferroptosis process, nanomaterials can improve therapeutic efficacy and demonstrate great promise in targeted therapy. A promising new treatment approach for OC is provided by this combo.

Zhang, Y. et al. created the nanomedicine known as SPIO-Serum, which is made up of superparamagnetic iron oxide nanoparticles attached to human serum. By increasing iron absorption, causing mitochondrial damage, and causing lipid peroxidation, the nanoparticles dramatically increase ferroptosis in OC cells (150). This finding offers a fresh theoretical foundation for iron oxide nanomaterial-based OC treatment. Furthermore, Cheng, S. et al. created a novel ferroptosis inducer using an ultrasound-responsive Bi2MoO6-MXene (BMO-MXene) heterojunction. By increasing ROS production and suppressing GPX4 and SLC7A11 expression, BMO-MXene markedly increased ferroptosis in OC cells. Subsequent research revealed that BMO-MXene may stimulate immunogenic cell death (ICD) when ultrasound is applied, which would activate the tumor immune microenvironment and stop OC from growing (151). This research offers a novel approach to the accurate and non-invasive management of OC. Li, G. et al. created HA@PFG nanoparticles based on hyaluronic acid in another study. By releasing Fe³^+^ into the OC tumor microenvironment, these nanoparticles triggered ferroptosis by causing lipid peroxidation and GSH consumption (152). Furthermore, by targeting the highly expressed cholesterol receptor SR-B1 in OC cells, HDL-like nanoparticles (HDL NPs) caused lipid peroxidation and ferroptosis, which blocked cholesterol absorption and decreased GPX4 expression. HDL NPs can greatly increase the killing effect of chemotherapy-resistant OC cells when combined with chemotherapeutic medications (like carboplatin) (153). This study targets SR-B1 and cholesterol metabolism, offering a novel approach to treating OC.

Iron Nitroprusside (FeNP) is an iron-based self-therapeutic nanomaterial that exerts significant anti-tumor effects in OC by inducing ferroptosis and chemodynamic therapy (CDT). In the acidic lysosomal environment, FeNP releases Fe²^+^, which catalyzes the Fenton reaction, converting endogenous H_2_O_2_ into ·OH. This generates ROS and lipid peroxides, triggering ferroptosis. Additionally, FeNP disrupts redox homeostasis by inhibiting GPX4 expression, further promoting ferroptosis (154).The development of novel nanomedicines offers an innovative therapeutic strategy for treating OC.

### 4.3 Application of small molecule compounds

Since the mechanism of ferroptosis has been gradually revealed, the use of small molecule drugs in the treatment of OC has shown great promise, particularly in causing ferroptosis, which offers a novel approach to OC treatment.

Erastin is a classic ferroptosis inducer. According to research by Zhan, S. et al., Erastin induces ferroptosis in OC cells by inhibiting xCT transporters and inducing the accumulation of ROS, and significantly enhances the sensitivity of OC cells to chemotherapeutic drugs such as cisplatin (155). In addition, Cang, W. et al. found that Erastin induces TAMs to polarize to M2 phenotype by activating the STAT3 signaling pathway and secreting IL-8, which significantly enhances the migration and invasion ability of ferroptosis-resistant OC cells (156). ABCB1 is a drug efflux transporter, and its overexpression is closely related to multidrug resistance in OC. Erastin can also enhance the sensitivity of ABCB1-overexpressing OC cells to Docetaxel by inhibiting the drug efflux activity of ABCB1 (157). In recent years, sodium molybdate (Na2MoO4) has steadily gained attention because of its potential for treating cancer (158, 159). Mao, G. et al. showed that Na2MoO4 significantly induced ferroptosis of OC cells by increasing the level of LIP, promoting the production of NO, and depleting GSH (160). Based on ferroptosis, the novel small molecule USP2 inhibitor ML364 has demonstrated anti-tumor potential (161). Yang, D. et al.showed that ML364 significantly enhanced RSL3-induced ferroptosis by downregulating Cyclin D1 and increasing ROS levels, providing new ideas for the development of ferroptosis-based therapeutic strategies (162). Another novel small molecule compound, linoleic acid-glucosamine hybrid (LA-GlcN), has the ability to enter tumor cells and selectively recognize overexpressed glucose transporter 1 (GLUT1), thereby inducing ferroptosis in HGSOC cells and offering a new approach to the precision treatment of high-grade serous OC (163). Mihajlović, E. et al. showed that FETPY, a novel diiron(I)sulfur-carbene complex, triggers ferroptosis in OC cells by promoting iron uptake and inducing membrane lipid peroxidation (164). Shen, X. et al. found that the platinum (II) complex PtQ induced ferroptosis in OC cells by downregulating the SLC7A11/GPX4 signaling axis and significantly enhancing lipid peroxidation and the accumulation of ROS (165). NTX-301 is a novel DNA demethylating agent. Wang, Y. et al. showed that NTX-301 enhances the ferroptosis sensitivity of OC cells by upregulating the expression of ACSL4. In addition, NTX-301 induces increased lipid peroxidation levels and further exacerbates cell ferroptosis by regulating iron metabolism-related genes (166). The identification of these small molecule compounds shows the potential clinical use of ferroptosis-based treatment for OC and offers a new anti-cancer method.

### 4.4 Increasing the usage of current medications

One of the key tactics in the treatment of cancer is the repurposing of current medications for new uses. A growing number of research have started to investigate the novel function of conventional medications in the treatment of cancer, particularly through the anti-tumor effects of ferroptosis, a novel mechanism of cell death (167, 168).

Ropivacaine is a local anesthetic drug that belongs to the amide anesthetic class. The amide anesthetic class includes the local anesthetic medication ropivacaine. Apart from its use in clinical anesthesia, research has demonstrated that ropivacaine causes ferroptosis by raising intracellular levels of ROS and free iron (Fe²^+^) and dramatically reduces the stemness of OC cells by blocking the PI3K/AKT signaling pathway. In addition to preventing cancer cells from proliferating, this form of action may also successfully overcome tumor stem cell resistance to treatment. The anti-tumor activity of ropivacaine was further confirmed by *in vivo* tests, which offered a theoretical foundation for its use in the treatment of OC (169). Meanwhile, there has been modest advancement in the repurposing of other widely used medications for the treatment of tumors. For instance, the sedative dexmedetomidine has been shown to control immunological responses in the tumor microenvironment and may potentially influence pathways linked to ferroptosis by causing oxidative stress (170). Furthermore, recent research has demonstrated that some medicines, such doxycycline, can also cause ferroptosis by disrupting lipid metabolism or triggering oxidative stress pathways, offering a possible novel therapy approach for OC (171, 172). Future advancements in the treatment of OC are anticipated to come from the expansion of the use of current medications by focusing on ferroptosis.

### 4.5 Natural products in the treatment of OC via targeting ferroptosis

As is shown in **Figure 6**, in recent years, natural products have become an important direction in cancer treatment research due to their wide range of biological activities and low side effects (**Table 1**). Several studies have shown that natural products have the potential to regulate the expression levels of ferroptosis-related factors (173). Natural products in particular are considered to be a potentially effective therapy option for OC. In the treatment of OC, natural products can not only promote the death of cancer cells by promoting ferroptosis, but also enhance the sensitivity of cancer cells to ferroptosis, forming a synergistic effect with existing therapies (such as chemotherapy, radiotherapy, etc.), thereby improving the treatment effect.

**Table 1:** Natural products in the treatment of OC via promoting ferroptosis.

Compound	Classification	Concentration	Model	Effect	Mechanism/target	Reference
Shikonin	Naphthoquinone Compounds	Shi: 0.8 mg/kg, Cis: 3.0 mg/kg (*in vivo*); Shi: 1.0-1.5 µM, Cis: 7-12 µM (*in vitro*)	Cisplatin-resistant OC cells (A2780/DDP, SKOV3/DDP, OVCAR4/DDP)	Ferroptosis↑, ROS↑, LPO↑, Fe²^+^↑, GPX4↓	HMOX1↑, TFRC↑, LTF↑, POR↑, NCOA4↑	(174)
Daphnetin	Coumarins	5-40 µg/ml	A2780, SKOV3, OVCAR8 (OC cells), IOSE80 (normal)	ROS↑, Apoptosis↑, Autophagy (cytoprotective)↑	AMPK↑, Akt/mTOR↓	(175)
Agrimonolide	Coumarins	10-40 µM (*in vitro*); 50 mg/kg (*in vivo*)	A2780, SKOV-3 cells; SKOV-3 xenograft model	Ferroptosis↑, ROS↑, Fe²^+^↑, SLC7A11↓, GPX4↓	Targets SCD1: inhibits SCD1 protein translation and stability	(176)
Curcumin and its derivatives	Polyphenols	IC50: 3.9 µM	Anglne/HO8910PM cells	Ferroptosis↑, GPX4↓, LPO↑, HCAR1↓, MCT1↓	HCAR1/MCT1↓, AMPK↑, SREBP1↓	(177)
Honokiol	Polyphenols	*In vitro*: 25-100 µM; *In vivo*: 30 mg/kg	SKOV3, 3AO cells; Xenograft mouse model	Ferroptosis↑, ROS↑, LPO↑, Fe²^+^↑, SLC7A11↓, GPX4↓	Binds to OTUB2, inhibits YAP signaling, promotes lipid peroxidation	(178)
Anisomycin	Antibiotic	31.8 µM	HuOCSCs	Ferroptosis↑, ATP↓, GSH↓, LPO↑, Fe²^+^↑	ATF4↓, GSH metabolism↓, Autophagy↓	(179)
Progesterone + Niraparib	Steroids	P4: 5 mg/kg, Nir: 50 mg/kg (*in vivo*); P4: 10 µM, Nir: 10 µM (*in vitro*)	OVCAR3, A2780, SKOV3 cells; Xenograft mouse model	Ferroptosis↑, ROS↑, LPO↑, GPX4↓, Fe²^+^↑	SCD1↑ mediates POA production, promotes lipid peroxidation	(180)
Sodium Selenite (SS)	Trace Elements and Derivatives	*In vitro*: >333 ng/mL; *In vivo*: 1000–2000 µg/kg	SKOV3 cells; SKOV3 xenograft mouse model	Ferroptosis↑, ROS↑, LPO↑, GPX4↓	GPX4↓, GSH depletion, Lipid peroxidation↑	(181)
Carboxymethylated Pachyman (CMP)	Polysaccharides	*In vitro*: 100-200 µg/ml; *In vivo*: 50 mg/kg	SKOV3, Hey cells; Xenograft mouse model	Ferroptosis↑, ROS↑, LPO↑, Fe²^+^↑, GSH↓	Downregulates Nrf2/HO-1/xCT/GPX4 pathway	(182)
Portulaca oleracea L. polysaccharide (POL)	Polysaccharides	*In vitro*: 50-100 µg/ml; *In vivo*: 50 mg/kg	SKOV3, Hey cells; Xenograft mouse model	Ferroptosis↑, ROS↑, LPO↑, Fe²^+^↑, GSH↓	Upregulates ACSL4, promotes lipid peroxidation	(183)
Angelica sinensis polysaccharide (ASP)	Polysaccharides	*In vitro*: 50-300 µg/mL; *In vivo*: 0.2 mg/kg/day	SKOV3/DDP, A2780/DDP cells; Xenograft mouse model	Ferroptosis↑, ROS↑, LPO↑, GSH↓, GPX4↓	Downregulates GPX4 expression, promotes lipid peroxidation	(184)
Laminarin	Polysaccharides	*In vitro*: 0.1–2 mg/mL; *In vivo*: 2 mg/mL	ES2, OV90 cells; Zebrafish xenograft model	Ferroptosis↑, ROS↑, LPO↑, Ca²^+^↑, MMP↓, PI3K/MAPK↓	Induces ER-mitochondrial calcium transfer, inhibits PI3K/MAPK signaling	(185)
Chelerythrine (CHE)	Alkaloids	*In vitro*: 5-15 µM; *In vivo*: 5–10 mg/kg	A2780, SKOV3 cells; Xenograft mouse model	Ferroptosis↑, ROS↑, LPO↑, Fe²^+^↑, GSH↓	Downregulates Nrf2/HO-1/GPX4 pathway	(186)
Chaetoglobosin K (ChK)	Alkaloids	*In vitro*: 1-10 µM; *In vivo*: N/A	OVCAR-3, A2780/CP70 cells; Normal ovarian IOSE-364 cells	Ferroptosis↑, ROS↑, LPO↑, GPX4↓, p53↑, Caspase-8↑	Activates p53, induces lipid peroxidation, G2 phase arrest via p38 MAPK	(187)
Harmine	Alkaloids	*In vitro*: 20-30 µM; *In vivo*: 60 mg/kg	SKOV3 cells; Xenograft mouse model	Ferroptosis↑, ROS↑, LPO↑, Fe²^+^↑, GSH↓, SOD↓	Downregulates SLC7A11/xCT, NRF2, GPX4	(188)
Artesunate (ART)	Terpenes	*In vitro*: 40 µM; *In vivo*: 70 mg/kg	SKOV3, ES2 cells; Xenograft mouse model	Ferroptosis↑, ROS↑, LPO↑, GSH↓, GPX4↓	Downregulates HOXC11, inhibits PROM2/PI3K/AKT	(189)
Costunolide	Terpenes	*In vitro*: 10-40 µM; *In vivo*: 5–10 mg/kg	SKOV3PT, A2780PT cells; Xenograft mouse model	Ferroptosis↑, ROS↑, LPO↑, GSH↓, Bcl-2↓	Induces ROS production, downregulates Bcl-2, inhibits NF-κB pathway	(190)
MAP30	Proteins and Peptides	*In vitro*: 10-40 µg/mL; *In vivo*: 125-250 µg/kg	ES2, A2780cp, OVCA433 cells; Xenograft mouse model	Ferroptosis↑, ROS↑, LPO↑, GSH↓, GPX4↓	Upregulates ROS, suppresses GPX4, inhibits AKT/ERK/FOXM1 and mTOR pathways	(191)
Norcantharidin (NCTD)	Terpenoids	*In vitro*: 10-20 µg/ml; *In vivo*: 100–200 mg/kg	SKOV3, OVCAR-3 cells; Xenograft mouse model	Ferroptosis↑, ROS↑, LPO↑, Fe²^+^↑, GSH↓, GPX4↓, xCT↓	Downregulates NRF2/HO-1/GPX4/xCT pathway	(192)
Tripterygium glycosides (TG)	Terpenoids	*In vitro*: 100-1600 µg/mL; *In vivo*: 1 mg/kg	A2780/DDP cells; Xenograft mouse model	Ferroptosis↑, ROS↑, LPO↑, GSH↓, Fe²^+^↑, GPX4↓	Inhibits NRF2/GPX4 pathway, reduces antioxidant defense	(193)
Obacunone (OB)	Terpenoids	*In vitro*: 10-40 µM; *In vivo*: 100 mg/kg	SKOV3, OVCAR3 cells; Xenograft mouse model	Ferroptosis↑, ROS↑, LPO↑, GSH↓, GPX4↓, ACSL4↑	Downregulates Akt phosphorylation, upregulates p53, promotes lipid peroxidation	(194)
δ-Tocotrienol	Terpenoids	*In vitro*: 5-20 µg/ml; *In vivo*: Not reported	IGROV-1, SKOV-3 cells (cisplatin-sensitive and resistant)	Ferroptosis↑, ROS↑, LPO↑, GPX4↓, JNK↑, p38↑	Induces oxidative stress, activates MAPK signaling, synergizes with cisplatin	(195)
SB-SD Extract	Traditional Chinese Medicine	*In vitro*: 50-600 µg/mL; *In vivo*: up to 250 µg/mL	A2780 cells; Zebrafish tumor model	Ferroptosis↑, ROS↑, LPO↑, GSH↓, Fe²^+^↑, FTH1↓	Induces heme catabolism and ferritinophagy, regulates NFE2L2/HMOX1/MAP1LC3B/NCOA4 pathway	(196)
Sodium citrate	trace elements and their derivatives	*In vitro*: 15–50 mM; *In vivo*: 4 g/kg/day	SKOV3, A2780 cells; Xenograft mouse model	Ferroptosis↑, Apoptosis↑, ROS↑, LPO↑, Fe²^+^↑, GPX4↓	Inhibits CAMKK2/AMPK pathway, activates NCOA4-mediated ferritinophagy	(197)
Eriodictyol	Flavonoids	*In vitro*: 10-50 µM; *In vivo*: Not reported	SKOV3 cells	Ferroptosis↑, ROS↑, LPO↑, GPX4↓, SLC7A11↓	Downregulates Nrf2/HO-1/NQO1, induces ferritinophagy	(198)

↑ represents upregulate, ↓ represents downregulate.

In this review, we summarized the roles of several natural products, including flavonoids, polyphenols, alkaloids, terpenes, polysaccharides, coumarins, proteins and peptides, trace elements and their derivatives, Traditional Chinese Medicine, naphthoquinone compounds, steroid compounds, etc., in OC and the mechanisms by which they regulate ferroptosis-related pathways to achieve their anti-cancer effects. We also analyzed their potential clinical application prospects, providing a new perspective for the development and application of natural products in the treatment of OC.

#### 4.5.1 Flavonoids

Eriodictyol is a natural flavonoid compound that regulates the Nrf2/HO-1/NQO1 signaling pathway, inhibits the expression of GPX4 and SLC7A11, thereby significantly enhancing the levels of intracellular ROS and LPO, and ultimately inducing ferroptosis. In addition, eriodictyol promotes mitochondrial dysfunction and ferritinophagy, exacerbating oxidative stress and effectively inhibiting the proliferation of OC cells (such as SKOV3), and its ferroptosis effect can be reversed by Ferrostatin-1 (198).

#### 4.5.2 Polyphenols

Curcumin is a natural polyphenolic compound derived from the rhizome of turmeric, which has been widely studied for cancer treatment in recent years (199). In an OC model, curcumin and its derivatives significantly induced ferroptosis by inhibiting the activity of GPX4 and downregulating the expression of key molecules such as MCT1 and HCAR1. Curcumin can enhance the generation of ROS and lipid peroxides, while causing lipid metabolism imbalance by inhibiting cellular antioxidant capacity. In addition, curcumin activated the AMPK signaling pathway and inhibited the SREBP1-mediated fatty acid synthesis pathway, further exacerbating the ferroptosis effect. *In vivo* studies have shown that the curcumin derivative NL01 can significantly inhibit the growth of OC transplanted tumors without causing obvious toxicity (177).

Honokiol is a biphenyl lignan extracted from the bark extract of Magnolia plant species, which has a wide range of antioxidant, antimicrobial, antidiabetic, anti-inflammatory, anti-aggregant, analgesic, antitumor, antiviral and neuroprotective activities (200). Honokiol inhibits the YAP signaling pathway by binding to the deubiquitinase OTUB2, significantly upregulating LPO and ROS, and inducing ferroptosis in OC cells. At the same time, Honokiol can reduce the expression of key antioxidant factors SLC7A11 and GPX4, further weakening the antioxidant capacity of tumor cells. Its *in vivo* experimental results show good safety and significant anti-tumor effects (178).

#### 4.5.3 Alkaloids

Harmine is a natural alkaloid derived from the plant Peganum harmala, which has anti-inflammatory, antibacterial, anti-diabetic, anti-malarial, anti-depressant and significant anti-tumor effects (201). Harmine significantly reduces the risk of OC. Studies have shown that Harmine significantly induces ferroptosis in OC cells SKOV3 by downregulating ferroptosis inhibitors such as SLC7A11/xCT, NRF2, and GPX4, promoting Fe²^+^ accumulation, LPO, and ROS generation. Additionally, Harmine also leads to a decrease in GSH and SOD levels, further exacerbating oxidative damage (188).

Chelerythrine (CHE) is a benzylisoquinoline alkaloid derived from plants. Studies have shown that CHE can trigger ferroptosis by downregulating the Nrf2/HO-1/GPX4 signaling pathway, reducing antioxidant defense capacity, and increasing lipid peroxidation and ROS accumulation. In addition, CHE significantly reduced intracellular GSH levels, accompanied by an increase in Fe²^+^. *In vitro* experiments showed that CHE could inhibit the proliferation of OC cells A2780 and SKOV3 and induce ferroptosis, while combined treatment with the Nrf2 activator TBHQ could reverse the CHE-induced ferroptosis effect. *In vivo*, CHE significantly inhibited tumor growth in the SKOV3 xenograft model and further enhanced the ferroptosis effect by inhibiting the expression of Nrf2 and related antioxidant proteins (186).

Anisomycin is an antibiotic purified from Streptomyces lividans and is a potential anticancer drug. Anisomycin weakens the cell’s ability to clear ROS and lipid peroxidation by inhibiting intracellular GSH levels and the activity of the key antioxidant enzyme GPX4, leading to the accumulation of lipid peroxides and the occurrence of cellular ferroptosis. In addition, Anisomycin can further aggravate the effects of ferroptosis by regulating the autophagy signaling pathway, and its mechanism of action is closely related to the inhibition of GSH metabolism mediated by ATF4. In the OC model, Anisomycin not only significantly promoted the accumulation of Fe²^+^, but also induced oxidative damage to cell membrane lipids, ultimately triggering ferroptosis signals (179).

Hydroquinidine (HQ) is a natural alkaloid derived from cinchona bark, which has significant anticancer effects in OC cells. HQ activates the oxidative stress pathway by inhibiting the expression of cell cycle-related proteins CDK1 and CDK6, significantly enhancing the apoptotic effect associated with ferroptosis. In addition, HQ can further aggravate the accumulation of intracellular lipid peroxides by reducing the expression level of GPX4 and inhibiting the activity of ferroportin. In SKOV-3 OC cells, HQ showed significant anti-proliferation, migration inhibition and apoptosis induction effects, and had low toxicity to normal cells, providing a new strategy for the treatment of OC (202).

Chaetoglobosin K (ChK) belongs to the class of natural products called mycotoxins and is also classified as an alkaloid, specifically an indole-diterpenoid alkaloid. This compound is extracted from fungi and has shown significant anticancer activity in platinum-resistant OC cells (203). ChK can inhibit cancer cell growth through multiple mechanisms, including inducing ferroptosis and apoptosis (204). Studies have shown that ChK triggers ferroptosis by downregulating the expression of the antioxidant factor GPX4 and increasing the levels of ROS and lipid peroxidation. In addition, ChK can further enhance cancer cell death by upregulating the p53 signaling pathway and activating the caspase-8-dependent extrinsic apoptosis pathway. Both *in vivo* and *in vitro* experiments have shown that ChK has low toxicity to normal ovarian cells, showing good selectivity and therapeutic potential (187).

#### 4.5.4 Terpenoids

Artesunate (ART) is a well-tolerated antimalarial drug and a potential anti-OC drug that induces ferroptosis (205). Artesunate has been widely studied. Studies have shown that (206) ART inhibits the transcription factor HOXC11, reduces the activity of the downstream PROM2/PI3K/AKT signaling pathway, significantly induces lipid peroxidation and ROS production, and reduces GSH levels, thereby triggering ferroptosis. In addition, ART further aggravates oxidative damage by inhibiting the expression of GPX4. Both *in vitro* and *in vivo* experiments have shown that ART significantly inhibits the proliferation, migration and growth of OC cells, and its effect of inducing ferroptosis can be partially reversed by overexpression of HOXC11 (189).

Costunolide is a natural sesquiterpene lactone compound that has shown significant inhibitory effects on platinum-resistant OC cells in research. Costunolide significantly increases the generation of ROS while reducing the level of antioxidant factor GSH, triggering lipid peroxidation and leading to cell death. Its induced ferroptosis mechanism is achieved by downregulating Bcl-2 protein, enhancing mitochondrial membrane permeability and inhibiting NF-κB pathway. In addition, costunolide also exhibits synergistic effects with cisplatin, effectively overcoming the chemotherapy barriers of platinum-resistant OC by promoting the accumulation of ROS and increasing the sensitivity of cells to cisplatin (190).

Obacunone (OB) is a natural bioactive compound extracted from citrus fruits. OB activates the Akt/p53 signaling pathway by downregulating Akt phosphorylation and upregulating p53 expression, thereby promoting iron-dependent lipid peroxidation and ROS production and inducing ferroptosis in OC cells. In addition, OB significantly reduces the levels of GSH and GPX4, while upregulating the expression of ACSL4, further enhancing the ferroptosis effect (194).

Norcantharidin (NCTD) is an effective anticancer drug. Studies have shown that NCTD is a potential anti-OC therapeutic agent, and its mechanism of action depends on the regulation of the NRF2/HO-1/GPX4/xCT axis, thereby promoting the accumulation of ROS, lipid peroxidation and Fe²^+^, inducing OC cell death. *In vivo* and *in vitro* experiments, NCTD not only significantly reduced the survival rate of OC cells, but also inhibited the growth of transplanted tumors, while significantly increasing the levels of ferroptosis-related markers MDA and PTGS2 (192).

Tripterygium glycosides (TG) is an active ingredient extracted from the traditional Chinese medicine Tripterygium wilfordii, which has anti-inflammatory, immunomodulatory and anti-cancer effects. Studies have shown that TG induces ferroptosis by regulating the NRF2/GPX4 signaling axis. TG treatment can significantly promote the accumulation of ROS and lipid peroxidation, while exacerbating the depletion of GSH and increasing the concentration of intracellular Fe²^+^. Studies have also shown that when TG is used in combination with cisplatin, it can significantly enhance the toxicity of cisplatin to drug-resistant OC cells (such as A2780/DDP) through a synergistic effect. *In vivo* studies have shown that TG alone or in combination with cisplatin significantly inhibited the growth of transplanted tumors and prolonged the survival time of tumor-bearing mice without showing obvious toxic side effects (193). Tripterygium wilfordii glycosides provide a potential new strategy for the treatment of OC resistance.

δ-Tocotrienol is a natural derivative of vitamin E and a type of terpenoid. δ-Tocotrienol is widely found in plants and exhibits unique antioxidant and anti-tumor activities. Studies have shown that δ-tocotrienol effectively inhibits the growth of OC cells (OC) by inducing ferroptosis. In addition, δ-tocotrienol activated JNK and p38 protein phosphorylation in the MAPK signaling pathway, further enhancing the oxidative stress-induced cell death response. In cisplatin-resistant OC cells (such as SKOV-3), δ-tocotrienol not only significantly reduced cell proliferation, but also synergized with cisplatin treatment to significantly improve the anti-cancer effect (195).

#### 4.5.5 Polysaccharides

Carboxymethylated Pachyman (CMP) is a polysaccharide derivative derived from Poria cocos. CMP weakens the antioxidant defense capacity of tumor cells by downregulating the Nrf2/HO-1/xCT/GPX4 signaling pathway, thereby inducing lipid peroxidation and ROS accumulation, and ultimately leading to ferroptosis. *In vitro* studies have shown that CMP significantly reduced GSH levels and increased the accumulation of Fe²^+^ and MDA in SKOV3 and Hey OC cells. At the same time, CMP significantly upregulated the expression of ferroptosis marker genes PTGS2 and CHAC1, and this effect could be reversed by the ferroptosis inhibitor Ferrostatin-1. *In vivo*, CMP significantly inhibited the growth of OC xenografts and reduced the expression levels of Nrf2, HO-1, xCT and GPX4 in tumor tissues (182).

Purslane polysaccharide is a natural polysaccharide derived from Purslane. Studies have shown that POL can significantly upregulate the expression of ACSL4, promote the accumulation of lipid peroxidation, ROS and Fe²^+^, and reduce the level of GSH, thereby inducing ferroptosis. In addition, the survival rate of OC cells decreased significantly after POL treatment, while the ferroptosis inhibitors Ferrostatin-1 and Deferoxamine were able to reverse this phenomenon, further proving that POL induces cell death through iron-dependent lipid peroxidation. *In vivo* studies also confirmed that POL can significantly inhibit the growth of OC xenografts, as manifested by a decrease in tumor volume and weight, and a significant increase in ACSL4, MDA and ROS levels in tumor tissues (183).

Angelica sinensis polysaccharide (ASP) is derived from the traditional Chinese medicine Angelica sinensis. Studies by Guo W et al. have shown that ASP significantly downregulates the expression level of the key antioxidant factor GPX4, weakens the antioxidant capacity of tumor cells, promotes the accumulation of ROS and lipid peroxidation, and thus enhances the ferroptosis effect. In addition, ASP exhibits a synergistic effect when used in combination with cisplatin, significantly inhibiting the proliferation, migration and invasion of cisplatin-resistant OC cells (such as SKOV3/DDP and A2780/DDP), while significantly increasing their apoptosis levels. *In vivo* studies have shown that the combination of ASP and cisplatin can significantly inhibit the growth of transplanted tumors, while having no obvious toxicity to normal tissues and organs, showing a good safety profile (184).

Laminarin is a natural polysaccharide derived from brown algae that achieves anti-cancer effects by inducing ferroptosis. Laminarin induces intracellular Ca²^+^ accumulation and loss of mitochondrial membrane potential by regulating the endoplasmic reticulum-mitochondrial axis-related proteins GRP75 and MFN2, significantly increasing ROS and lipid peroxidation levels. In addition, Laminarin also significantly reduces the proliferation ability of OC cells by inhibiting the phosphorylation of the PI3K/MAPK signaling pathway, and induces the synergistic effect of apoptosis and ferroptosis. In animal experiments, Laminarin showed significant anti-tumor activity in the zebrafish transplant tumor model, and no obvious toxicity was observed (185).

#### 4.5.6 Coumarins

Daphnetin is a natural coumarin compound. Ma N et al. showed that Daphnetin can inhibit NQO1 activity, leading to intracellular iron accumulation and lipid peroxidation, thereby activating ferroptosis, a non-apoptotic cell death pathway (207). Fan X et al.’s research showed that Daphnetin can also trigger cell death by inducing the generation of ROS and induce protective autophagy by regulating the AMPK/Akt/mTOR pathway (175). Ferroptosis mechanism provides theoretical support for the potential application of Daphnetin in the treatment of OC.

Agrimonolide is a natural isocoumarin product derived from the traditional Chinese medicinal herb Agrimonia pilosa Ledeb and has multiple functional activities (208). Liu Y et al.’s study showed that vanilloids significantly induced ferroptosis in OC cells A2780 and SKOV-3 by targeting and inhibiting the expression of SCD1. Specifically, vanilloids treatment increased intracellular ROS, total iron, and Fe²^+^ levels, and reduced the expression of ferroptosis marker proteins SLC7A11 and GPX4. SCD1 overexpression can significantly reverse the ferroptosis effect induced by vanilloids. In *in vivo* studies, vanilloids inhibited the growth of OC xenografts in a dose-dependent manner, while reducing the expression level of SCD1 in tumor tissues (176).

#### 4.5.7 Protein and peptide compounds

Momotarin (MAP30) is a natural active protein extracted from the seeds of Momordica charantia, which has antioxidant, anti-inflammatory, anti-cancer, anti-diabetic, anti-bacterial, anti-obesity and immunomodulatory activities (209). Momordica protein is a potential drug for adjuvant therapy of OC (210). Research by Chan DW et al. showed that (191)MAP30 significantly increased the Ca²^+^ concentration in OC cells. This change led to oxidative stress and mitochondrial dysfunction, further inducing the accumulation of ROS and lipid peroxidation. The experiment also found that the ferroptosis-inducing effect of MAP30 could be partially reversed by the ferroptosis inhibitor Ferrostatin-1. In addition, MAP30 can significantly inhibit the proliferation and migration of OC cells by inhibiting the AKT/ERK/FOXM1 and mTOR signaling pathways. MAP30 is a safe and effective natural product that can be used as a potential drug for the adjuvant treatment of OC.

#### 4.5.8 Trace elements and their derivatives

Sodium citrate is a naturally derived citrate. Sodium citrate chelates intracellular Ca²^+^, significantly reducing Ca²^+^ concentrations, thereby inhibiting the activity of the Ca²^+^/CAMKK2/AMPK signaling pathway. On the one hand, this mechanism induces cell apoptosis by inhibiting the HIF1α-dependent glycolysis pathway; on the other hand, sodium citrate triggers ferroptosis by activating NCOA4-mediated ferritinophagy, leading to iron ion (Fe²^+^) accumulation and increased lipid peroxidation levels. Studies have also found that sodium citrate can significantly enhance the sensitivity of OC cells to chemotherapeutic drugs, especially cisplatin and carboplatin (197).

Selenium (Se) is a trace element widely present in the natural environment and is also one of the important components of natural products. Studies have shown that high-dose sodium selenite (SS) significantly induces ferroptosis of OC cells by promoting ROS generation, weakening GPX4 expression, and inducing lipid peroxidation. In addition, *in vivo* experiments have verified that high-dose Se can significantly inhibit the growth of SKOV3 xenograft tumors and exhibit tumor-specific toxicity (181).

#### 4.5.9 Traditional Chinese medicine

Scutellaria barbata D. Don and Scleromitrion diffusum (SB-SD) is a traditional Chinese medicine combination for the treatment of OC. SB-SD extract significantly increases intracellular free iron (Fe²^+^),ROS, and lipid peroxidation levels by promoting heme catabolism and ferritinophagy, while reducing GSH and mitochondrial membrane potential, ultimately leading to cell death. Multi-omics analysis found that SB-SD forms a complex ferroptosis regulatory network by upregulating the expression of NFE2L2, HMOX1, MAP1LC3B, and NCOA4, and downregulating the expression of FTH1. In addition, the anti-tumor effect of SB-SD has been verified in both *in vivo* (zebrafish tumor model) and *in vitro* experiments. SB-SD is a potential natural anti-cancer treatment option (196).

#### 4.5.10 Naphthoquinones

Shikonin is a natural naphthoquinone compound derived from Lithospermum erythrorhizon. Studies have shown that shikonin can effectively overcome the resistance of OC to cisplatin by inducing ferroptosis. In cisplatin-resistant OC cells (such as A2780/DDP, SKOV3/DDP, and OVCAR4/DDP), the combination of shikonin and cisplatin significantly enhanced the level of cellular ferroptosis, including ROS generation, lipid peroxidation accumulation, and increased Fe²^+^. Its mechanism of action is related to the upregulation of HMOX1, which produces Fe²^+^ by promoting heme degradation, thereby exacerbating ROS generation and lipid peroxidation. In addition, shikonin also regulates the expression levels of other ferroptosis-related proteins (such as TFRC, LTF, POR, NCOA4) and key enzymes (such as GPX4) (174).

#### 4.5.11 Steroids

Progesterone is derived from steroidal natural products and is usually produced *in vivo* through cholesterol metabolism. Progesterone significantly promotes ferroptosis of OC cells by enhancing the production of POA and lipid oxidation. Studies have shown that progesterone can activate the expression of SCD1, a gene related to fatty acid metabolism, increase the expression of GPX4, and weaken the cell’s ability to clear lipid peroxides. In addition, progesterone combined with the PARP inhibitor Niraparib can further enhance DNA damage, leading to increased ROS and aggravated ferroptosis. *In vivo* studies have shown that this combined treatment can significantly inhibit the growth of OC transplanted tumors and prolong the survival of mice (180).

## 5 Limitations and considerations for future research directions

Ferroptosis is a regulated form of cell death characterized by iron accumulation and uncontrolled lipid peroxidation. This leads to plasma membrane disruption and the release of intracellular contents. Initially studied as a targeted therapy for cancer cells with oncogenic RAS mutations, ferroptosis induction now shows potential to complement chemotherapy, immunotherapy, and radiotherapy in various cancer types. However, it can cause side effects, including immune cell death, bone marrow damage, liver and kidney damage, cachexia (severe weight loss and muscle wasting), and secondary tumorigenesis (211). Currently, most studies on ferroptosis focus on animal models and cell lines, and there are significant differences between different species. Although mouse models and human cell lines provide preliminary evidence for the potential of ferroptosis in the treatment of OC, there is still uncertainty as to whether their results can accurately reflect the clinical situation of human patients. For example, the expression levels and mechanisms of key molecules in the ferroptosis pathway, such as FSP1 and GPX4, may vary between species (212–214). Therefore, validation using more clinically relevant animal models and human samples is critical in future studies.

Although some laboratory research progress has been made in ferroptosis as a new target for the treatment of OC, there are still challenges in translating these findings into effective clinical treatments. The regulatory pathways of ferroptosis are relatively complex and may involve multiple cross-signaling pathways, such as ROS generation, lipid peroxidation, autophagy, inflammatory response, etc. In clinical studies, the regulation of these mechanisms may be affected by factors such as individual differences among patients and tumor heterogeneity. Therefore, future studies need more clinical data to verify the actual effect of ferroptosis-targeted therapy and optimize treatment strategies. Ferroptosis is not an isolated cell death pathway. Studies have found that it interacts with other cell death pathways such as apoptosis, necrosis, and pyroptosis (97). For example, ferroptosis may work together with apoptosis pathways or be regulated by other cell death pathways in some cases. Therefore, future studies need to explore the interactions between these cell death pathways in more depth and consider jointly targeting multiple cell death pathways to improve the therapeutic effect. Although targeted therapy for ferroptosis has shown potential in laboratory studies, OC cells may develop resistance in clinical treatment. The resistance mechanism may be related to factors such as the regulation of key molecules in the ferroptosis pathway, the balance of ROS generation, and changes in iron metabolism. Future studies need to reveal the resistance mechanism associated with ferroptosis and explore how to overcome this resistance to achieve more lasting therapeutic effects. Individual differences in ferroptosis regulation may affect its effectiveness as a therapeutic target. Different OC patients may respond differently to ferroptosis inducers due to differences in gene mutations, tumor microenvironment, immune response, and other factors. Therefore, the development of personalized treatment strategies is critical. Future studies can explore the use of multidimensional data such as genomics and proteomics to analyze the response of OC patients to ferroptosis therapy, so as to develop personalized treatment plans.

At present, drug development for ferroptosis is still in its infancy, and the development of targeted drugs is progressing slowly. The diversity and complexity of ferroptosis regulatory molecules pose huge challenges to drug development. Future research should focus on developing more efficient and selective ferroptosis inducers and solving problems such as drug biocompatibility, dose control, and targeting. In clinical applications, physiological conditions such as liver and kidney function of patients should also be considered to avoid treatment-related side effects. Although ferroptosis, as an emerging target for the treatment of OC, has been supported by laboratory and small-scale preclinical studies, it is still necessary to verify its efficacy and safety in extensive clinical trials. Large-scale, long-term clinical trials are needed in the future to evaluate the true effect of ferroptosis-targeted therapy in the treatment of OC and provide strong data support for drug approval.

## 6 Discussions

This review systematically summarizes the research progress on ferroptosis in OC treatment, focusing particularly on its potential to overcome chemotherapy resistance and enhance treatment efficacy. Targeting ferroptosis can effectively inhibit the growth, metastasis, and drug resistance of OC cells, while improving chemotherapy outcomes. Ferroptosis plays a crucial role in the onset, progression, and metastasis of OC. OC cells often have a high dependence on iron metabolism, which makes them more prone to ferroptosis than normal cells. Key mechanisms of ferroptosis include lipid peroxidation, ROS accumulation, and membrane rupture. Molecular pathways such as GPX4, SLC7A11, and ACSL4 are central in regulating this process. By targeting these key molecules, and by modulating iron metabolism and antioxidant defense systems, ferroptosis can be induced directly to kill OC cells and overcome chemotherapy resistance. Research on natural products, small molecule compounds, and novel nanomedicines has opened new avenues for clinical ferroptosis application. Natural products like rutin, curcumin, and magnolol can induce ferroptosis and enhance OC cells’ sensitivity to chemotherapy by regulating antioxidant factors and lipid metabolism. Small molecules, such as Erastin and RSL3, further promote ferroptosis by inhibiting critical factors like GPX4 and SLC7A11. The development of novel nanomedicines offers targeted treatment options, especially in combination with ferroptosis mechanisms, to improve treatment efficacy and overcome drug resistance.

Despite its promising potential, ferroptosis-based therapy faces several challenges. First, efficient induction of ferroptosis still suffers from selectivity issues. While ferroptosis can selectively target tumor cells, the lack of fully specific ferroptosis inducers remains a limitation. Ensuring accurate control over ferroptosis induction and minimizing damage to normal tissues is an ongoing challenge. Second, individual variation in iron metabolism, antioxidant capacity, and ferroptosis sensitivity among OC patients complicates the individualized regulation of ferroptosis-related molecules. As a result, future ferroptosis therapies will need more personalized approaches. Moreover, optimizing the combination of ferroptosis inducers with other treatment modalities, such as chemotherapy or targeted therapies, warrants further investigation. Future research should focus on the following areas: first, deepen understanding of ferroptosis regulation to improve its selectivity and therapeutic effectiveness; second, develop more selective ferroptosis inducers and explore their combination with existing chemotherapy or targeted therapies; third, identify biomarkers for ferroptosis-related molecules to guide individualized treatment strategies; last but not least, investigate the synergistic effects of ferroptosis with other treatments, such as immunotherapy and radiotherapy, to provide more comprehensive treatment options for OC.

In conclusion, ferroptosis represents a promising therapeutic target for OC, with great potential for clinical application. By advancing our understanding of its mechanisms, developing more selective ferroptosis inducers, and optimizing combination therapies, we hope to provide OC patients with more precise and effective treatment options in the future.

## References

[1] Webb PM Jordan SJ. Global epidemiology of epithelial ovarian cancer. Nat Rev Clin Oncol. (2024) 21:389–400. doi: 10.1038/s41571-024-00881-3 38548868

[2] Rodriguez IV Ghezelayagh T Pennington KP Norquist BM. Prevention of ovarian cancer: where are we now and where are we going? Curr Oncol Rep. (2024) 26:1355–66. doi: 10.1007/s11912-024-01587-6 39115678

[3] Webb PM Green AC Jordan SJ. Trends in hormone use and ovarian cancer incidence in US white and Australian women: implications for the future. Cancer Causes Control CCC. (2017) 28:365–70. doi: 10.1007/s10552-017-0868-0 28233113

[4] Mize BK Salvi A Ren Y Burdette JE Fuchs JR. Discovery and development of botanical natural products and their analogues as therapeutics for ovarian cancer. Nat Prod Rep. (2023) 40:1250–70. doi: 10.1039/d2np00091a PMC10448539 37387219

[5] Li X Li Z Ma H Li X Zhai H Li X. Ovarian cancer: Diagnosis and treatment strategies (Review). Oncol Lett. (2024) 28:441. doi: 10.3892/ol.2024.14574 39099583 PMC11294909

[6] Dixon SJ Lemberg KM Lamprecht MR Skouta R Zaitsev EM Gleason CE. Ferroptosis: an iron-dependent form of nonapoptotic cell death. Cell. (2012) 149:1060–72. doi: 10.1016/j.cell.2012.03.042 PMC3367386 22632970

[7] Grzelak MM Chmura Ł Wróbel PM Adamek D Lankosz M Jach R. Investigation of the role and chemical form of iron in the ovarian carcinogenesis process. J Trace Elem Med Biol. (2020) 60:126500. doi: 10.1016/j.jtemb.2020.126500 32203723

[8] Hou K Liu L Fang Z-H Zong W-X Sun D Guo Z. The role of ferroptosis in cardio-oncology. Arch Toxicol. (2024) 98:709–34. doi: 10.1007/s00204-023-03665-3 PMC12931676 38182913

[9] Bhatia T Doshi G Godad A. PARP inhibitors in ovarian cancer: Mechanisms, resistance, and the promise of combination therapy. Pathol - Res Pract. (2024) 263:155617. doi: 10.1016/j.prp.2024.155617 39357181

[10] Huang Z Ma Y Sun Z Cheng L Wang G. Ferroptosis: potential targets and emerging roles in pancreatic diseases. Arch Toxicol. (2024) 98:75–94. doi: 10.1007/s00204-023-03625-x 37934210

[11] Ćwiertnia A Kozłowski M Cymbaluk-Płoska A. The role of iron and cobalt in gynecological diseases. Cells. (2022) 12:117. doi: 10.3390/cells12010117 36611913 PMC9818544

[12] Wang Y Wu X Ren Z Li Y Zou W Chen J. Overcoming cancer chemotherapy resistance by the induction of ferroptosis. Drug Resist Update. (2023) 66:100916. doi: 10.1016/j.drup.2022.100916 36610291

[13] Nie Z Chen M Gao Y Huang D Cao H Peng Y. Ferroptosis and tumor drug resistance: current status and major challenges. Front Pharmacol. (2022) 13:879317. doi: 10.3389/fphar.2022.879317 35668934 PMC9163417

[14] Lu B Chen XB Ying MD He QJ Cao J Yang B. The role of ferroptosis in cancer development and treatment response. Front Pharmacol. (2018) 8:992. doi: 10.3389/fphar.2017.00992 29375387 PMC5770584

[15] Ren Y Mao X Xu H Dang Q Weng S Zhang Y. Ferroptosis and EMT: key targets for combating cancer progression and therapy resistance. Cell Mol Life Sci. (2023) 80:263. doi: 10.1007/s00018-023-04907-4 37598126 PMC10439860

[16] Zuo S Yu J Pan H Lu L. Novel insights on targeting ferroptosis in cancer therapy. biomark Res. (2020) 8:50. doi: 10.1186/s40364-020-00229-w 33024562 PMC7532638

[17] Li D Geng D Wang M. Advances in natural products modulating autophagy influenced by cellular stress conditions and their anticancer roles in the treatment of ovarian cancer. FASEB J. (2024) 38:e70075. doi: 10.1096/fj.202401409R 39382031

[18] Zhang X Wei X Shi L Jiang H Ma F Li Y. The latest research progress: Active components of traditional Chinese medicine as promising candidates for ovarian cancer therapy. J Ethnopharmacol. (2025) 337:118811. doi: 10.1016/j.jep.2024.118811 39251149

[19] Zhou J Jiang Y-Y Wang H-P Chen H Wu Y-C Wang L. Natural compound Tan-I enhances the efficacy of Paclitaxel chemotherapy in ovarian cancer. Ann Transl Med. (2020) 8:752–2. doi: 10.21037/atm-20-4072 PMC7333144 32647677

[20] Zhou L Xu T Zhang Y Zhu M Zhu W Wang Z. Transcriptional network in ovarian cancer cell line SKOV3 treated with pinellia pedatisecta schott extract. Oncol Rep. (2016) 36:462–70. doi: 10.3892/or.2016.4779 27176137

[21] Zhou J Jiang Y-Y Wang X-X Wang H-P Chen H Wu Y-C. Tanshinone IIA suppresses ovarian cancer growth through inhibiting Malignant properties and angiogenesis. Ann Transl Med. (2020) 8:1295–5. doi: 10.21037/atm-20-5741 PMC7661888 33209875

[22] Dixon SJ Stockwell BR. The role of iron and reactive oxygen species in cell death. Nat Chem Biol. (2014) 10:9–17. doi: 10.1038/nchembio.1416 24346035

[23] Gaschler MM Stockwell BR. Lipid peroxidation in cell death. Biochem Biophys Res Commun. (2017) 482:419–25. doi: 10.1016/j.bbrc.2016.10.086 PMC5319403 28212725

[24] Sourkes T. The discovery of lecithin, the first phospholipid.

[25] Spector AA Kim H-Y. Discovery of essential fatty acids. J Lipid Res. (2015) 56:11–21. doi: 10.1194/jlr.R055095 25339684 PMC4274059

[26] Sies H. Oxidative stress: A concept in redox biology and medicine. Redox Biol. (2015) 4:180–3. doi: 10.1016/j.redox.2015.01.002 PMC4309861 25588755

[27] Forman HJ Zhang H. Targeting oxidative stress in disease: Promise and limitations of antioxidant therapy. Nat Rev Drug Discov. (2021) 20:689–709. doi: 10.1038/s41573-021-00233-1 34194012 PMC8243062

[28] Yang WS Stockwell BR. Ferroptosis: Death by lipid peroxidation. Trends Cell Biol. (2016) 26:165–76. doi: 10.1016/j.tcb.2015.10.014 PMC4764384 26653790

[29] Stockwell BR Jiang X. The chemistry and biology of ferroptosis. Cell Chem Biol. (2020) 27:365–75. doi: 10.1016/j.chembiol.2020.03.013 PMC7204503 32294465

[30] Yang S Hu C Chen X Tang Y Li J Yang H. Crosstalk between metabolism and cell death in tumorigenesis. Mol Cancer. (2024) 23:71. doi: 10.1186/s12943-024-01977-1 38575922 PMC10993426

[31] Jiang X Stockwell BR Conrad M. Ferroptosis: Mechanisms, biology and role in disease. Nat Rev Mol Cell Biol. (2021) 22:266–82. doi: 10.1038/s41580-020-00324-8 PMC8142022 33495651

[32] Li D Zhang M Chao H. Significance of glutathione peroxidase 4 and intracellular iron level in ovarian cancer cells-”utilization” of ferroptosis mechanism. Inflammation Res Off J Eur Histamine Res Soc Al. (2021) 70:1177–89. doi: 10.1007/s00011-021-01495-6 34537856

[33] Chen F Kang R Tang D Liu J. Ferroptosis: principles and significance in health and disease. J Hematol OncolJ Hematol Oncol. (2024) 17:41. doi: 10.1186/s13045-024-01564-3 38844964 PMC11157757

[34] Endale HT Tesfaye W Mengstie TA. ROS induced lipid peroxidation and their role in ferroptosis. Front Cell Dev Biol. (2023) 11:1226044. doi: 10.3389/fcell.2023.1226044 37601095 PMC10434548

[35] Li R Yuan H Zhang C Han D Wang Y Feng L. Induced ferroptosis pathway by regulating cellular lipid peroxidation with peroxynitrite generator for reversing “cold” tumors. Small Weinh Bergstr Ger. (2024) 20:e2404807. doi: 10.1002/smll.202404807 39279600

[36] Wang B Wang Y Zhang J Hu C Jiang J Li Y. ROS-induced lipid peroxidation modulates cell death outcome: Mechanisms behind apoptosis, autophagy, and ferroptosis. Arch Toxicol. (2023) 97:1439–51. doi: 10.1007/s00204-023-03476-6 37127681

[37] Akiyama H Carter BZ Andreeff M Ishizawa J. Molecular mechanisms of ferroptosis and updates of ferroptosis studies in cancers and leukemia. Cells. (2023) 12:1128. doi: 10.3390/cells12081128 37190037 PMC10136912

[38] Pope LE Dixon SJ. Regulation of ferroptosis by lipid metabolism. Trends Cell Biol. (2023) 33:1077–87. doi: 10.1016/j.tcb.2023.05.003 PMC10733748 37407304

[39] Lu C Zhou X Zhang L. Phospholipids with two polyunsaturated fatty acyl tails: An important driver of ferroptosis. MedComm. (2024) 5:e606. doi: 10.1002/mco2.606 38919333 PMC11196922

[40] Qiu B Zandkarimi F Bezjian CT Reznik E Soni RK Gu W. Phospholipids with two polyunsaturated fatty acyl tails promote ferroptosis. Cell. (2024) 187:1177–1190.e18. doi: 10.1016/j.cell.2024.01.030 38366593 PMC10940216

[41] Liu J Kang R Tang D. Signaling pathways and defense mechanisms of ferroptosis. FEBS J. (2022) 289:7038–50. doi: 10.1111/febs.16059 34092035

[42] Lin J Lai Y Lu F Wang W. Targeting ACSLs to modulate ferroptosis and cancer immunity. Trends Endocrinol Metab. (2024). doi: 10.1016/j.tem.2024.09.003 39424456

[43] Stockwell BR Friedmann Angeli JP Bayir H Bush AI Conrad M Dixon SJ. Ferroptosis: A regulated cell death nexus linking metabolism, redox biology, and disease. Cell. (2017) 171:273–85. doi: 10.1016/j.cell.2017.09.021 PMC5685180 28985560

[44] Cui J Wang Y Tian X Miao Y Ma L Zhang C. LPCAT3 is transcriptionally regulated by YAP/ZEB/EP300 and collaborates with ACSL4 and YAP to determine ferroptosis sensitivity. Antioxid Redox Signal. (2023) 39:491–511. doi: 10.1089/ars.2023.0237 37166352

[45] Mashima R Okuyama T. The role of lipoxygenases in pathophysiology; new insights and future perspectives. Redox Biol. (2015) 6:297–310. doi: 10.1016/j.redox.2015.08.006 26298204 PMC4556770

[46] Pandey AV Flück CE. NADPH P450 oxidoreductase: Structure, function, and pathology of diseases. Pharmacol Ther. (2013) 138:229–54. doi: 10.1016/j.pharmthera.2013.01.010 23353702

[47] Zou Y Li H Graham ET Deik AA Eaton JK Wang W. Cytochrome P450 oxidoreductase contributes to phospholipid peroxidation in ferroptosis. Nat Chem Biol. (2020) 16:302–9. doi: 10.1038/s41589-020-0472-6 PMC7353921 32080622

[48] Kraft VAN Bezjian CT Pfeiffer S Ringelstetter L Müller C Zandkarimi F. GTP cyclohydrolase 1/tetrahydrobiopterin counteract ferroptosis through lipid remodeling. ACS Cent Sci. (2020) 6:41–53. doi: 10.1021/acscentsci.9b01063 31989025 PMC6978838

[49] Vasquez-Vivar J Shi Z Tan S. Tetrahydrobiopterin in cell function and death mechanisms. Antioxid Redox Signal. (2022) 37:171–83. doi: 10.1089/ars.2021.0136 PMC9293684 34806400

[50] Patanè GT Putaggio S Tellone E Barreca D Ficarra S Maffei C. Ferroptosis: Emerging role in diseases and potential implication of bioactive compounds. Int J Mol Sci. (2023) 24:17279. doi: 10.3390/ijms242417279 38139106 PMC10744228

[51] Dlouhy AC Bailey DK Steimle BL Parker HV Kosman DJ. Fluorescence resonance energy transfer links membrane ferroportin, hephaestin but not ferroportin, amyloid precursor protein complex with iron efflux. J Biol Chem. (2019) 294:4202–14. doi: 10.1074/jbc.RA118.005142 PMC6422103 30647129

[52] Cao H Schroeder B Chen J Schott MB McNiven MA. The endocytic fate of the transferrin receptor is regulated by c-abl kinase. J Biol Chem. (2016) 291:16424–37. doi: 10.1074/jbc.M116.724997 PMC4974358 27226592

[53] Oosterheert W van Bezouwen LS Rodenburg RNP Granneman J Förster F Mattevi A. Cryo-EM structures of human STEAP4 reveal mechanism of iron(III) reduction. Nat Commun. (2018) 9:4337. doi: 10.1038/s41467-018-06817-7 30337524 PMC6194020

[54] Torti SV Manz DH Paul BT Blanchette-Farra N Torti FM. Iron and cancer. Annu Rev Nutr. (2018) 38:97–125. doi: 10.1146/annurev-nutr-082117-051732 30130469 PMC8118195

[55] Habashy HO Powe DG Staka CM Rakha EA Ball G Green AR. Transferrin receptor (CD71) is a marker of poor prognosis in breast cancer and can predict response to tamoxifen. Breast Cancer Res Treat. (2010) 119:283–93. doi: 10.1007/s10549-009-0345-x 19238537

[56] Katsarou A Pantopoulos K. Basics and principles of cellular and systemic iron homeostasis. Mol Aspects Med. (2020) 75:100866. doi: 10.1016/j.mam.2020.100866 32564977

[57] Brookes MJ Hughes S Turner FE Reynolds G Sharma N Ismail T. Modulation of iron transport proteins in human colorectal carcinogenesis. Gut. (2006) 55:1449–60. doi: 10.1136/gut.2006.094060 PMC1856421 16641131

[58] Chen X Kang R Kroemer G Tang D. Broadening horizons: the role of ferroptosis in cancer. Nat Rev Clin Oncol. (2021) 18:280–96. doi: 10.1038/s41571-020-00462-0 33514910

[59] Mou Y Wang J Wu J He D Zhang C Duan C. Ferroptosis, a new form of cell death: Opportunities and challenges in cancer. J Hematol OncolJ Hematol Oncol. (2019) 12:34. doi: 10.1186/s13045-019-0720-y 30925886 PMC6441206

[60] Lyamzaev KG Huan H Panteleeva AA Simonyan RA Avetisyan AV Chernyak BV. Exogenous iron induces mitochondrial lipid peroxidation, lipofuscin accumulation, and ferroptosis in H9c2 cardiomyocytes. Biomolecules. (2024) 14:730. doi: 10.3390/biom14060730 38927133 PMC11201805

[61] Kang R Kroemer G Tang D. The tumor suppressor protein p53 and the ferroptosis network. Free Radic Biol Med. (2019) 133:162–8. doi: 10.1016/j.freeradbiomed.2018.05.074 PMC6251771 29800655

[62] Fantone S Piani F Olivieri F Rippo MR Sirico A Di Simone N. Role of SLC7A11/xCT in ovarian cancer. Int J Mol Sci. (2024) 25:587. doi: 10.3390/ijms25010587 38203758 PMC10779187

[63] Ou Y Wang S-J Li D Chu B Gu W. Activation of SAT1 engages polyamine metabolism with p53-mediated ferroptotic responses. Proc Natl Acad Sci U.S.A. (2016) 113:E6806–12. doi: 10.1073/pnas.1607152113 PMC5098629 27698118

[64] Xu R Wang W Zhang W. Ferroptosis and the bidirectional regulatory factor p53. Cell Death Discov. (2023) 9:197. doi: 10.1038/s41420-023-01517-8 37386007 PMC10310766

[65] Chen D Chu B Yang X Liu Z Jin Y Kon N. iPLA2β-mediated lipid detoxification controls p53-driven ferroptosis independent of GPX4. Nat Commun. (2021) 12:3644. doi: 10.1038/s41467-021-23902-6 34131139 PMC8206155

[66] Li J-Y Feng Y-H Li Y-X He P-Y Zhou Q-Y Tian Y-P. Ferritinophagy: A novel insight into the double-edged sword in ferritinophagy-ferroptosis axis and human diseases. Cell Prolif. (2024) 57:e13621. doi: 10.1111/cpr.13621 38389491 PMC11216947

[67] HuangGCaiYRenMZhangXFuYChengR. Salidroside sensitizes triple-negative breast cancer to ferroptosis by SCD1-mediated lipogenesis and NCOA4-mediated ferritinophagy. J Adv Res. (2024). doi: 10.1016/j.jare.2024.09.027 39353532

[68] Zhou B Liu J Kang R Klionsky DJ Kroemer G Tang D. Ferroptosis is a type of autophagy-dependent cell death. Semin Cancer Biol. (2020) 66:89–100. doi: 10.1016/j.semcancer.2019.03.002 30880243

[69] Liu M Fan Y Li D Han B Meng Y Chen F. Ferroptosis inducer erastin sensitizes NSCLC cells to celastrol through activation of the ROS-mitochondrial fission-mitophagy axis. Mol Oncol. (2021) 15:2084–105. doi: 10.1002/1878-0261.12936 PMC8334255 33675143

[70] Lee S Hwang N Seok BG Lee S Lee S-J Chung SW. Autophagy mediates an amplification loop during ferroptosis. Cell Death Dis. (2023) 14:464. doi: 10.1038/s41419-023-05978-8 37491375 PMC10368698

[71] Lee Y-S Lee D-H Choudry HA Bartlett DL Lee YJ. Ferroptosis-induced endoplasmic reticulum stress: Cross-talk between ferroptosis and apoptosis. Mol Cancer Res MCR. (2018) 16:1073–6. doi: 10.1158/1541-7786.MCR-18-0055 PMC6030493 29592897

[72] Ji X Chen Z Lin W Wu Q Wu Y Hong Y. Esculin induces endoplasmic reticulum stress and drives apoptosis and ferroptosis in colorectal cancer via PERK regulating eIF2α/CHOP and Nrf2/HO-1 cascades. J Ethnopharmacol. (2024) 328:118139. doi: 10.1016/j.jep.2024.118139 38561058

[73] Tak J Kim YS Kim TH Park G-C Hwang S Kim SG. Gα12 overexpression in hepatocytes by ER stress exacerbates acute liver injury via ROCK1-mediated miR-15a and ALOX12 dysregulation. Theranostics. (2022) 12:1570–88. doi: 10.7150/thno.67722 PMC8825599 35198058

[74] Cheng R Wang X Huang L Lu Z Wu A Guo S. Novel insights into the protective effects of leonurine against acute kidney injury: Inhibition of ER stress-associated ferroptosis via regulating ATF4/CHOP/ACSL4 pathway. Chem Biol Interact. (2024) 395:111016. doi: 10.1016/j.cbi.2024.111016 38670420

[75] Zhong P Li L Feng X Teng C Cai W Zheng W. Neuronal ferroptosis and ferroptosis-mediated endoplasmic reticulum stress: Implications in cognitive dysfunction induced by chronic intermittent hypoxia in mice. Int Immunopharmacol. (2024) 138:112579. doi: 10.1016/j.intimp.2024.112579 38944951

[76] He F Zhang P Liu J Wang R Kaufman RJ Yaden BC. ATF4 suppresses hepatocarcinogenesis by inducing SLC7A11 (xCT) to block stress-related ferroptosis. J Hepatol. (2023) 79:362–77. doi: 10.1016/j.jhep.2023.03.016 PMC11332364 36996941

[77] Ruan D Wen J Fang F Lei Y Zhao Z Miao Y. Ferroptosis in epithelial ovarian cancer: a burgeoning target with extraordinary therapeutic potential. Cell Death Discov. (2023) 9:434. doi: 10.1038/s41420-023-01721-6 38040696 PMC10692128

[78] Liu M Wu K Wu Y. The emerging role of ferroptosis in female reproductive disorders. BioMed Pharmacother Biomed Pharmacother. (2023) 166:115415. doi: 10.1016/j.biopha.2023.115415 37660655

[79] Kapper C Oppelt P Arbeithuber B Gyunesh AA Vilusic I Stelzl P. Targeting ferroptosis in ovarian cancer: Novel strategies to overcome chemotherapy resistance. Life Sci. (2024) 349:122720. doi: 10.1016/j.lfs.2024.122720 38762066

[80] Liu D Hu Z Lu J Yi C. Redox-regulated iron metabolism and ferroptosis in ovarian cancer: molecular insights and therapeutic opportunities. Antioxidants. (2024) 13:791. doi: 10.3390/antiox13070791 39061859 PMC11274267

[81] Beddows I Fan H Heinze K Johnson BK Leonova A Senz J. Cell state of origin impacts development of distinct endometriosis-related ovarian carcinoma histotypes. Cancer Res. (2024) 84:26–38. doi: 10.1158/0008-5472.CAN-23-1362 37874327 PMC10758692

[82] Battaglia AM Sacco A Perrotta ID Faniello MC Scalise M Torella D. Iron administration overcomes resistance to erastin-mediated ferroptosis in ovarian cancer cells. Front Oncol. (2022) 12:868351. doi: 10.3389/fonc.2022.868351 35433479 PMC9008715

[83] Wang C-K Chen T-J Tan GYT Chang F-P Sridharan S Yu C-HA. MEX3A mediates p53 degradation to suppress ferroptosis and facilitate ovarian cancer tumorigenesis. Cancer Res. (2023) 83:251–63. doi: 10.1158/0008-5472.CAN-22-1159 PMC9845988 36354374

[84] Xuan Y Wang H Yung MM Chen F Chan W-S Chan Y-S. SCD1/FADS2 fatty acid desaturases equipoise lipid metabolic activity and redox-driven ferroptosis in ascites-derived ovarian cancer cells. Theranostics. (2022) 12:3534–52. doi: 10.7150/thno.70194 PMC9065188 35547771

[85] Wang Y Hu M Cao J Wang F Han JR Wu TW. ACSL4 and polyunsaturated lipids support metastatic extravasation and colonization. Cell. (2024). doi: 10.1016/j.cell.2024.10.047 39591965

[86] Xu K Zheng X Shi H Ou J Ding H. MAD2L2, a key regulator in ovarian cancer and promoting tumor progression. Sci Rep. (2024) 14:130. doi: 10.1038/s41598-023-50744-7 38167649 PMC10761867

[87] Liu Y Li J Xu J Long Y Wang Y Liu X. m6A-driven NAT10 translation facilitates fatty acid metabolic rewiring to suppress ferroptosis and promote ovarian tumorigenesis through enhancing ACOT7 mRNA acetylation. Oncogene. (2024) 43:3498–516. doi: 10.1038/s41388-024-03185-z 39390256

[88] Li X-X Xiong L Wen Y Zhang Z-J. Comprehensive analysis of the tumor microenvironment and ferroptosis-related genes predict prognosis with ovarian cancer. Front Genet. (2021) 12:774400. doi: 10.3389/fgene.2021.774400 34868262 PMC8634641

[89] Li Y Gong X Hu T Chen Y. Two novel prognostic models for ovarian cancer respectively based on ferroptosis and necroptosis. BMC Cancer. (2022) 22:74. doi: 10.1186/s12885-021-09166-9 35039008 PMC8764839

[90] Wang H Cheng Q Chang K Bao L Yi X. Integrated analysis of ferroptosis-related biomarker signatures to improve the diagnosis and prognosis prediction of ovarian cancer. Front Cell Dev Biol. (2021) 9:807862. doi: 10.3389/fcell.2021.807862 35071242 PMC8766510

[91] Wei C Zhao G Gao M Liu Y Lei P Cao T. Construction of an immunity and ferroptosis-related risk score model to predict ovarian cancer clinical outcomes and immune microenvironment. Front Biosci Landmark Ed. (2023) 28:4. doi: 10.31083/j.fbl2801004 36722270

[92] Gao J Pang X Ren F Zhu L. Identification of a ferroptosis-related long non-coding RNA signature for prognosis prediction of ovarian cancer. Carcinogenesis. (2023) 44:80–92. doi: 10.1093/carcin/bgac082 36300656

[93] Ji H-Z Chen L Ren M Li S Liu T-Y Chen H-J. CXCL8 promotes endothelial-to-mesenchymal transition of endothelial cells and protects cells from erastin-induced ferroptosis via CXCR2-mediated activation of the NF-κB signaling pathway. Pharm Basel Switz. (2023) 16:1210. doi: 10.3390/ph16091210 PMC10536478 37765018

[94] Wang Y Zhang H Zhan Y Li Z Li S Guo S. Comprehensive in silico analysis of prognostic and immune infiltrates for FGFs in human ovarian cancer. J Ovarian Res. (2024) 17:197. doi: 10.1186/s13048-024-01496-z 39385288 PMC11465590

[95] Li Z Liao X Hu Y Li M Tang M Zhang S. SLC27A4-mediated selective uptake of mono-unsaturated fatty acids promotes ferroptosis defense in hepatocellular carcinoma. Free Radic Biol Med. (2023) 201:41–54. doi: 10.1016/j.freeradbiomed.2023.03.013 36924851

[96] Atiya HI Frisbie L Goldfeld E Orellana T Donnellan N Modugno F. Endometriosis-associated mesenchymal stem cells support ovarian clear cell carcinoma through iron regulation. Cancer Res. (2022) 82:4680–93. doi: 10.1158/0008-5472.CAN-22-1294 PMC9755968 36219681

[97] Zhang C Liu N. Ferroptosis, necroptosis, and pyroptosis in the occurrence and development of ovarian cancer. Front Immunol. (2022) 13:920059. doi: 10.3389/fimmu.2022.920059 35958626 PMC9361070

[98] Chen J Wei Z Fu K Duan Y Zhang M Li K. Non-apoptotic cell death in ovarian cancer: Treatment, resistance and prognosis. BioMed Pharmacother Biomed Pharmacother. (2022) 150:112929. doi: 10.1016/j.biopha.2022.112929 35429741

[99] Zhang Q Li N Deng L Jiang X Zhang Y Lee LTO. ACSL1-induced ferroptosis and platinum resistance in ovarian cancer by increasing FSP1 N-myristylation and stability. Cell Death Discov. (2023) 9:83. doi: 10.1038/s41420-023-01385-2 36882396 PMC9992462

[100] Zhang Y Xia F Liu X Yu Z Xie L Liu L. JAM3 maintains leukemia-initiating cell self-renewal through LRP5/AKT/β-catenin/CCND1 signaling. J Clin Invest. (2018) 128:1737–51. doi: 10.1172/JCI93198 PMC5919829 29584620

[101] Yamaguchi M Hirai S Idogawa M Sumi T Uchida H Fujitani N. Junctional adhesion molecule 3 is a potential therapeutic target for small cell lung carcinoma. Exp Cell Res. (2023) 426:113570. doi: 10.1016/j.yexcr.2023.113570 36990421

[102] Wang N Chen M Wu M Liao Y Xia Q Cai Z. High-adhesion ovarian cancer cell resistance to ferroptosis: The activation of NRF2/FSP1 pathway by junctional adhesion molecule JAM3. Free Radic Biol Med. (2024) 228:1–13. doi: 10.1016/j.freeradbiomed.2024.12.040 39706500

[103] Atwani R Nagare RP Rogers A Prasad M Lazar V Sandusky G. Integrin-linked kinase-frizzled 7 interaction maintains cancer stem cells to drive platinum resistance in ovarian cancer. J Exp Clin Cancer Res CR. (2024) 43:156. doi: 10.1186/s13046-024-03083-y 38822429 PMC11143768

[104] Cabarca S Ili C Vanegas C Gil L Vertel-Morrinson M Brebi P. Drug resistance biomarkers in ovarian cancer: a bibliometric study from 2017 to 2022. Front Oncol. (2024) 14:1450675. doi: 10.3389/fonc.2024.1450675 39588300 PMC11586235

[105] Wang Y Zhao G Condello S Huang H Cardenas H Tanner EJ. Frizzled-7 identifies platinum-tolerant ovarian cancer cells susceptible to ferroptosis. Cancer Res. (2021) 81:384–99. doi: 10.1158/0008-5472.CAN-20-1488 PMC7855035 33172933

[106] Zhao L Yang H Wang Y Yang S Jiang Q Tan J. STUB1 suppresses paclitaxel resistance in ovarian cancer through mediating HOXB3 ubiquitination to inhibit PARK7 expression. Commun Biol. (2024) 7:1439. doi: 10.1038/s42003-024-07127-z 39501077 PMC11538469

[107] Wu X Shen S Qin J Fei W Fan F Gu J. High co-expression of SLC7A11 and GPX4 as a predictor of platinum resistance and poor prognosis in patients with epithelial ovarian cancer. Bjog- Int J Obstet Gynaecol. (2022) 129 Suppl 2:40–9. doi: 10.1111/1471-0528.17327 PMC10108211 36485069

[108] Yusuf RZ Saez B Sharda A van Gastel N Yu VWC Baryawno N. Aldehyde dehydrogenase 3a2 protects AML cells from oxidative death and the synthetic lethality of ferroptosis inducers. Blood. (2020) 136:1303–16. doi: 10.1182/blood.2019001808 PMC7483435 32458004

[109] Dong H He L Sun Q Zhan J Li J Xiong X. Inhibit ALDH3A2 reduce ovarian cancer cells survival via elevating ferroptosis sensitivity. Gene. (2023) 876:147515. doi: 10.1016/j.gene.2023.147515 37247796

[110] Zhang S Zheng F Zhang L Huang Z Huang X Pan Z. LncRNA HOTAIR-mediated MTHFR methylation inhibits 5-fluorouracil sensitivity in esophageal cancer cells. J Exp Clin Cancer Res CR. (2020) 39:131. doi: 10.1186/s13046-020-01610-1 32653028 PMC7353690

[111] Woloszynska-Read A Zhang W Yu J Link PA Mhawech-Fauceglia P Collamat G. Coordinated cancer germline antigen promoter and global DNA hypomethylation in ovarian cancer: Association with the BORIS/CTCF expression ratio and advanced stage. Clin Cancer Res Off J Am Assoc Cancer Res. (2011) 17:2170–80. doi: 10.1158/1078-0432.CCR-10-2315 PMC3079045 21296871

[112] Wang X Xu Z Ren X Chen X Yi Q Zeng S. MTHFR inhibits TRC8-mediated HMOX1 ubiquitination and regulates ferroptosis in ovarian cancer. Clin Transl Med. (2022) 12:e1013. doi: 10.1002/ctm2.1013 36149747 PMC9505752

[113] Furutake Y Yamaguchi K Yamanoi K Kitamura S Takamatsu S Taki M. YAP1 suppression by ZDHHC7 is associated with ferroptosis resistance and poor prognosis in ovarian clear cell carcinoma. Mol Cancer Ther. (2024) 23:1652–65. doi: 10.1158/1535-7163.MCT-24-0145 38958503

[114] Zhang X Zheng X Ying X Xie W Yin Y Wang X. CEBPG suppresses ferroptosis through transcriptional control of SLC7A11 in ovarian cancer. J Transl Med. (2023) 21:334. doi: 10.1186/s12967-023-04136-0 37210575 PMC10199564

[115] Li C Wang Z Wang Y Liu H Cheng Y. MiR-93-5p inhibits ovarian cancer through SLC7A11-mediated-ferroptosis. Heliyon. (2024) 10:e35457. doi: 10.1016/j.heliyon.2024.e35457 39165989 PMC11334884

[116] Sun D Li Y-C Zhang X-Y. Lidocaine promoted ferroptosis by targeting miR-382-5p/SLC7A11 axis in ovarian and breast cancer. Front Pharmacol. (2021) 12:681223. doi: 10.3389/fphar.2021.681223 34122108 PMC8188239

[117] Ma L-L Liang L Zhou D Wang S-W. Tumor suppressor miR-424-5p abrogates ferroptosis in ovarian cancer through targeting ACSL4. Neoplasma. (2021) 68:165–73. doi: 10.4149/neo_2020_200707N705 33038905

[118] Wang K Mei S Cai M Zhai D Zhang D Yu J. Ferroptosis-related long noncoding RNAs as prognostic biomarkers for ovarian cancer. Front Oncol. (2022) 12:888699. doi: 10.3389/fonc.2022.888699 35756659 PMC9218568

[119] Cao L Wang Y Liu J Bai X Chi X. Long non-coding RNA TPT1-AS1 inhibits ferroptosis in ovarian cancer by regulating GPX4 via CREB1 regulation. Am J Reprod Immunol N Y N 1989. (2024) 92:e13864. doi: 10.1111/aji.13864 39141012

[120] Cai L Hu X Ye L Bai P Jie Y Shu K. Long non-coding RNA ADAMTS9-AS1 attenuates ferroptosis by targeting microRNA-587/solute carrier family 7 member 11 axis in epithelial ovarian cancer. Bioengineered. (2022) 13:8226–39. doi: 10.1080/21655979.2022.2049470 PMC9161843 35311457

[121] Chai B Wu Y Yang H Fan B Cao S Zhang X. Tau aggregation-dependent lipid peroxide accumulation driven by the hsa_circ_0001546/14-3-3/CAMK2D/tau complex inhibits epithelial ovarian cancer peritoneal metastasis. Adv Sci Weinh Baden-Wurtt Ger. (2024) 11:e2310134. doi: 10.1002/advs.202310134 PMC11186043 38634567

[122] Chen J Ye M Bai J Hu C Lu F Gu D. Novel insights into the interplay between m6A modification and programmed cell death in cancer. Int J Biol Sci. (2023) 19:1748–63. doi: 10.7150/ijbs.81000 PMC10092764 37063421

[123] Shi J-X Zhang Z-C Yin H-Z Piao X-J Liu C-H Liu Q-J. RNA m6A modification in ferroptosis: Implications for advancing tumor immunotherapy. Mol Cancer. (2024) 23:213. doi: 10.1186/s12943-024-02132-6 39342168 PMC11437708

[124] Pu X Wu Y Ji Q Fu S Zuo H Chu L. Mechanisms of N6−methyladenosine modification in tumor development and potential therapeutic strategies (review). Int J Oncol. (2023) 62:75. doi: 10.3892/ijo.2023.5523 37203412 PMC10198710

[125] Lou Y Huang K Xu B Chen X. METTL14 plays an oncogenic role in NSCLC by modulating ferroptosis and the m6A modification of GPX4. Arch Physiol Biochem. (2024) 130:962–73. doi: 10.1080/13813455.2024.2376813 38993012

[126] Tao X Kang N Zheng Z Zhu Z Ma J He W. The regulatory mechanisms of N6-methyladenosine modification in ferroptosis and its implications in disease pathogenesis. Life Sci. (2024) 355:123011. doi: 10.1016/j.lfs.2024.123011 39181316

[127] Li Y Guo M Qiu Y Li M Wu Y Shen M. Autophagy activation is required for N6-methyladenosine modification to regulate ferroptosis in hepatocellular carcinoma. Redox Biol. (2024) 69:102971. doi: 10.1016/j.redox.2023.102971 38056309 PMC10749285

[128] Dodson M de la Vega MR Cholanians AB Schmidlin CJ Chapman E Zhang DD. Modulating NRF2 in disease: timing is everything. Annu Rev Pharmacol Toxicol. (2019) 59:555–75. doi: 10.1146/annurev-pharmtox-010818-021856 PMC6538038 30256716

[129] Anandhan A Dodson M Shakya A Chen J Liu P Wei Y. NRF2 controls iron homeostasis and ferroptosis through HERC2 and VAMP8. Sci Adv. (2023) 9:eade9585. doi: 10.1126/sciadv.ade9585 36724221 PMC9891695

[130] Sang X Han J Wang Z Cai W Liao X Kong Z. SGK1 suppresses ferroptosis in ovarian cancer via NRF2-dependent and -independent pathways. Oncogene. (2024) 43:3335–47. doi: 10.1038/s41388-024-03173-3 39306614

[131] Li M Li L Cheng X Li L Tu K. Hypoxia promotes the growth and metastasis of ovarian cancer cells by suppressing ferroptosis via upregulating SLC2A12. Exp Cell Res. (2023) 433:113851. doi: 10.1016/j.yexcr.2023.113851 37940066

[132] Xiang H Wang M Chen Y-F Wu H-M Li M-G Guo L. Regulation of cancer cell ferroptosis by PTRF/cavin-1. Free Radic Res. (2024) 58:417–29. doi: 10.1080/10715762.2024.2386457 39079051

[133] Luo Y Liu X Chen Y Tang Q He C Ding X. Targeting PAX8 sensitizes ovarian cancer cells to ferroptosis by inhibiting glutathione synthesis. Apopt Int J Program Cell Death. (2024) 29:1499–514. doi: 10.1007/s10495-024-01985-y 38853202

[134] Xu S Liu Y Yang S Fei W Qin J Lu W. FXN targeting induces cell death in ovarian cancer stem-like cells through PRDX3-mediated oxidative stress. iScience. (2024) 27:110506. doi: 10.1016/j.isci.2024.110506 39184439 PMC11342215

[135] Miyahara S Ohuchi M Nomura M Hashimoto E Soga T Saito R. FDX2, an iron-sulfur cluster assembly factor, is essential to prevent cellular senescence, apoptosis or ferroptosis of ovarian cancer cells. J Biol Chem. (2024) 300:107678. doi: 10.1016/j.jbc.2024.107678 39151727 PMC11414659

[136] Hou R Sun X Cao S Wang Y Jiang L. Stabilization of SQLE mRNA by WTAP/FTO/IGF2BP3-dependent manner in HGSOC: Implications for metabolism, stemness, and progression. Cell Death Dis. (2024) 15:872. doi: 10.1038/s41419-024-07257-6 39617776 PMC11609299

[137] He J Siu MKY Ngan HYS Chan KKL. Aberrant cholesterol metabolism in ovarian cancer: Identification of novel therapeutic targets. Front Oncol. (2021) 11:738177. doi: 10.3389/fonc.2021.738177 34820325 PMC8606538

[138] Zhang R Zhang L Fan S Wang L Wang B Wang L. Squalene monooxygenase (SQLE) protects ovarian cancer cells from ferroptosis. Sci Rep. (2024) 14:22646. doi: 10.1038/s41598-024-72506-9 39349544 PMC11442994

[139] Han Y Fu L Kong Y Jiang C Huang L Zhang H. STEAP3 affects ovarian cancer progression by regulating ferroptosis through the p53/SLC7A11 pathway. Mediators Inflammation. (2024) 2024:4048527. doi: 10.1155/2024/4048527 PMC10911874 38440354

[140] Ebrahimi B Viswanadhapalli S Pratap UP Rahul G Yang X Pitta Venkata P. Pharmacological inhibition of the LIF/LIFR autocrine loop reveals vulnerability of ovarian cancer cells to ferroptosis. NPJ Precis Oncol. (2024) 8:118. doi: 10.1038/s41698-024-00612-y 38789520 PMC11126619

[141] Wang Y Wang S Zhang W. HRD1 functions as a tumor suppressor in ovarian cancer by facilitating ubiquitination-dependent SLC7A11 degradation. Cell Cycle Georget Tex. (2023) 22:1116–26. doi: 10.1080/15384101.2023.2178102 PMC10081055 36809917

[142] Yang W-H Lin C-C Wu J Chao P-Y Chen K Chen P-H. The hippo pathway effector YAP promotes ferroptosis via the E3 ligase SKP2. Mol Cancer Res MCR. (2021) 19:1005–14. doi: 10.1158/1541-7786.MCR-20-0534 PMC8178191 33707306

[143] Sinha BK Murphy C Brown SM Silver BB Tokar EJ Bortner CD. Mechanisms of cell death induced by erastin in human ovarian tumor cells. Int J Mol Sci. (2024) 25:8666. doi: 10.3390/ijms25168666 39201357 PMC11355013

[144] Yue W Yupeng G Jun C Kui J. Apatinib combined with olaparib induces ferroptosis via a p53-dependent manner in ovarian cancer. J Cancer Res Clin Oncol. (2023) 149:8681–9. doi: 10.1007/s00432-023-04811-1 PMC11796833 37120435

[145] Azumi M Kusama K Yoshie M Nakano S Tsuru A Kato T. Involvement of ferroptosis in eribulin-induced cytotoxicity in ovarian clear cell carcinoma. Eur J Pharmacol. (2024) 971:176544. doi: 10.1016/j.ejphar.2024.176544 38552939

[146] Hong T Lei G Chen X Li H Zhang X Wu N. PARP inhibition promotes ferroptosis via repressing SLC7A11 and synergizes with ferroptosis inducers in BRCA-proficient ovarian cancer. Redox Biol. (2021) 42:101928. doi: 10.1016/j.redox.2021.101928 33722571 PMC8113041

[147] Tang S Shen Y Wei X Shen Z Lu W Xu J. Olaparib synergizes with arsenic trioxide by promoting apoptosis and ferroptosis in platinum-resistant ovarian cancer. Cell Death Dis. (2022) 13:826. doi: 10.1038/s41419-022-05257-y 36163324 PMC9513087

[148] Xie X Chen C Wang C Guo Y Sun B Tian J. Targeting GPX4-mediated ferroptosis protection sensitizes BRCA1-deficient cancer cells to PARP inhibitors. Redox Biol. (2024) 76:103350. doi: 10.1016/j.redox.2024.103350 39265497 PMC11415882

[149] Heiserman JP Minhas Z Nikpayam E Cheon D-J. Targeting heat shock protein 27 and fatty acid oxidation augments cisplatin treatment in cisplatin-resistant ovarian cancer cell lines. Int J Mol Sci. (2023) 24:12638. doi: 10.3390/ijms241612638 37628819 PMC10454186

[150] Zhang Y Xia M Zhou Z Hu X Wang J Zhang M. p53 promoted ferroptosis in ovarian cancer cells treated with human serum incubated-superparamagnetic iron oxides. Int J Nanomed. (2021) 16:283–96. doi: 10.2147/IJN.S282489 PMC7811475 33469287

[151] Cheng S Zhou T Luo Y Zhang J Dong K Zhang Q. Ultrasound-responsive Bi2MoO6-MXene heterojunction as ferroptosis inducers for stimulating immunogenic cell death against ovarian cancer. J Nanobiotechnol. (2024) 22:408. doi: 10.1186/s12951-024-02658-3 PMC11238442 38992664

[152] Li G Shi S Tan J He L Liu Q Fang F. Highly efficient synergistic chemotherapy and magnetic resonance imaging for targeted ovarian cancer therapy using hyaluronic acid-coated coordination polymer nanoparticles. Adv Sci Weinh Baden-Wurtt Ger. (2024) 11:e2309464. doi: 10.1002/advs.202309464 PMC11538696 39287149

[153] Wang Y Calvert AE Cardenas H Rink JS Nahotko D Qiang W. Nanoparticle targeting in chemo-resistant ovarian cancer reveals dual axis of therapeutic vulnerability involving cholesterol uptake and cell redox balance. Adv Sci Weinh Baden-Wurtt Ger. (2024) 11:e2305212. doi: 10.1002/advs.202305212 PMC10987123 38263873

[154] Asif K Adeel M Rahman MM Caligiuri I Perin T Cemazar M. Iron nitroprusside as a chemodynamic agent and inducer of ferroptosis for ovarian cancer therapy. J Mater Chem B. (2023) 11:3124–35. doi: 10.1039/d2tb02691k 36883303

[155] Zhan S Yung MMH Siu MKY Jiao P Ngan HYS Chan DW. New insights into ferroptosis initiating therapies (FIT) by targeting the rewired lipid metabolism in ovarian cancer peritoneal metastases. Int J Mol Sci. (2022) 23:15263. doi: 10.3390/ijms232315263 36499591 PMC9737695

[156] Cang W Wu A Gu L Wang W Tian Q Zheng Z. Erastin enhances metastatic potential of ferroptosis-resistant ovarian cancer cells by M2 polarization through STAT3/IL-8 axis. Int Immunopharmacol. (2022) 113:109422. doi: 10.1016/j.intimp.2022.109422 36410184

[157] Zhou H-H Chen X Cai L-Y Nan X-W Chen J-H Chen X-X. Erastin reverses ABCB1-mediated docetaxel resistance in ovarian cancer. Front Oncol. (2019) 9:1398. doi: 10.3389/fonc.2019.01398 31921655 PMC6930896

[158] Manchester DK Gordon SK Golas CL Roberts EA Okey AB. Ah receptor in human placenta: Stabilization by molybdate and characterization of binding of 2,3,7,8-tetrachlorodibenzo-p-dioxin, 3-methylcholanthrene, and benzo(a)pyrene. Cancer Res. (1987) 47:4861–8. 3040233

[159] El-Waseif EG Sharawy MH Suddek GM. The modulatory effect of sodium molybdate against cisplatin-induced CKD: Role of TGF-β/smad signaling pathway. Life Sci. (2022) 306:120845. doi: 10.1016/j.lfs.2022.120845 35917941

[160] Mao G Xin D Wang Q Lai D. Sodium molybdate inhibits the growth of ovarian cancer cells via inducing both ferroptosis and apoptosis. Free Radic Biol Med. (2022) 182:79–92. doi: 10.1016/j.freeradbiomed.2022.02.023 35219846

[161] Yi J Tavana O Li H Wang D Baer RJ Gu W. Targeting USP2 regulation of VPRBP-mediated degradation of p53 and PD-L1 for cancer therapy. Nat Commun. (2023) 14:1941. doi: 10.1038/s41467-023-37617-3 37024504 PMC10079682

[162] Yang D Liu X Yang Y Long Y Nan D Shi B. Pharmacological USP2 targeting suppresses ovarian cancer growth by potentiating apoptosis and ferroptosis. Arch Biochem Biophys. (2024) 762:110193. doi: 10.1016/j.abb.2024.110193 39486565

[163] Chen Y Liao X Jing P Hu L Yang Z Yao Y. Linoleic acid-glucosamine hybrid for endogenous iron-activated ferroptosis therapy in high-grade serous ovarian cancer. Mol Pharm. (2022) 19:3187–98. doi: 10.1021/acs.molpharmaceut.2c00333 35939328

[164] Mihajlović E Biancalana L Jelača S Chiaverini L Dojčinović B Dunđerović D. FETPY: A diiron(I) thio-carbyne complex with prominent anticancer activity *in vitro* and in *vivo* . J Med Chem. (2024) 67:7553–68. doi: 10.1021/acs.jmedchem.4c00377 38639401

[165] Shen X Peng Y Zhou H Ye X Han Z Shi X. A pt(II) complex bearing N-heterocycle ring induced ferroptotic cell death in ovarian cancer. J Inorg Biochem. (2024) 253:112502. doi: 10.1016/j.jinorgbio.2024.112502 38335582

[166] Wang Y Situ X Cardenas H Siu E Alhunayan SA Keathley R. Preclinical evaluation of NTX-301, a novel DNA hypomethylating agent in ovarian cancer. Clin Cancer Res Off J Am Assoc Cancer Res. (2024) 30:1175–88. doi: 10.1158/1078-0432.CCR-23-2368 PMC10947827 38231483

[167] Gonzalez-Fierro A Dueñas-González A. Drug repurposing for cancer therapy, easier said than done. Semin Cancer Biol. (2021) 68:123–31. doi: 10.1016/j.semcancer.2019.12.012 31877340

[168] Xia Y Sun M Huang H Jin W-L. Drug repurposing for cancer therapy. Signal Transduct Target Ther. (2024) 9:92. doi: 10.1038/s41392-024-01808-1 38637540 PMC11026526

[169] Lu Y Mao J Xu Y Pan H Wang Y Li W. Ropivacaine represses the ovarian cancer cell stemness and facilitates cell ferroptosis through inactivating the PI3K/AKT signaling pathway. Hum Exp Toxicol. (2022) 41:9603271221120652. doi: 10.1177/09603271221120652 36124980

[170] Gao X Wang X-L. Dexmedetomidine promotes ferroptotic cell death in gastric cancer via hsa_circ_0008035/miR-302a/E2F7 axis. Kaohsiung J Med Sci. (2023) 39:390–403. doi: 10.1002/kjm2.12650 36718915 PMC11895938

[171] Tourville A Viguier S González-Lizárraga F Tomas-Grau RH Ramirez P Brunel J-M. Rescue of dopamine neurons from iron-dependent ferroptosis by doxycycline and demeclocycline and their non-antibiotic derivatives. Antioxid Basel Switz. (2023) 12:575. doi: 10.3390/antiox12030575 PMC10045597 36978822

[172] Crissey MAS Versace A Bhardwaj M Jain V Liu S Singh A. Divergent effects of acute and chronic PPT1 inhibition in melanoma. Autophagy. (2024) 1–13. doi: 10.1080/15548627.2024.2403152 PMC11760279 39265628

[173] Koeberle SC Kipp AP Stuppner H Koeberle A. Ferroptosis-modulating small molecules for targeting drug-resistant cancer: Challenges and opportunities in manipulating redox signaling. Med Res Rev. (2023) 43:614–82. doi: 10.1002/med.21933 PMC10947485 36658724

[174] Ni M Zhou J Zhu Z Xu Q Yin Z Wang Y. Shikonin and cisplatin synergistically overcome cisplatin resistance of ovarian cancer by inducing ferroptosis via upregulation of HMOX1 to promote Fe2+ accumulation. Phytomed Int J Phytother Phytopharm. (2023) 112:154701. doi: 10.1016/j.phymed.2023.154701 36773431

[175] Fan X Xie M Zhao F Li J Fan C Zheng H. Daphnetin triggers ROS-induced cell death and induces cytoprotective autophagy by modulating the AMPK/akt/mTOR pathway in ovarian cancer. Phytomed Int J Phytother Phytopharm. (2021) 82:153465. doi: 10.1016/j.phymed.2021.153465 33486268

[176] Liu Y Liu X Wang H Ding P Wang C. Agrimonolide inhibits cancer progression and induces ferroptosis and apoptosis by targeting SCD1 in ovarian cancer cells. Phytomed Int J Phytother Phytopharm. (2022) 101:154102. doi: 10.1016/j.phymed.2022.154102 35526323

[177] Shi M Zhang M-J Yu Y Ou R Wang Y Li H. Curcumin derivative NL01 induces ferroptosis in ovarian cancer cells via HCAR1/MCT1 signaling. Cell Signal. (2023) 109:110791. doi: 10.1016/j.cellsig.2023.110791 37406786

[178] Liu F Zhang Y Xia X Han J Cao L. Honokiol induces ferroptosis in ovarian cancer cells through the regulation of YAP by OTUB2. J Obstet Gynaecol Res. (2024) 50:864–72. doi: 10.1111/jog.15922 38480480

[179] Xiong Y Liu T Chen J. Anisomycin has the potential to induce human ovarian cancer stem cell ferroptosis by influencing glutathione metabolism and autophagy signal transduction pathways. J Cancer. (2023) 14:1202–15. doi: 10.7150/jca.83355 PMC10197939 37215446

[180] Wu N Zhang X Fang C Zhu M Wang Z Jian L. Progesterone enhances niraparib efficacy in ovarian cancer by promoting palmitoleic-acid-mediated ferroptosis. Res Wash DC. (2024) 7:371. doi: 10.34133/research.0371 PMC11116976 38798714

[181] Choi J-A Lee EH Cho H Kim J-H. High-dose selenium induces ferroptotic cell death in ovarian cancer. Int J Mol Sci. (2023) 24:1918. doi: 10.3390/ijms24031918 36768241 PMC9915545

[182] Jing T Guo Y Wei Y. Carboxymethylated pachyman induces ferroptosis in ovarian cancer by suppressing NRF1/HO-1 signaling. Oncol Lett. (2022) 23:161. doi: 10.3892/ol.2022.13281 35399331 PMC8987927

[183] Xia L Yang M Liu Y. Portulaca oleracea L. polysaccharide inhibits ovarian cancer via inducing ACSL4-dependent ferroptosis. Aging. (2024) 16:5108–22. doi: 10.18632/aging.205608 PMC11006488 38503553

[184] Guo W Wang W Lei F Zheng R Zhao X Gu Y. Angelica sinensis polysaccharide combined with cisplatin reverses cisplatin resistance of ovarian cancer by inducing ferroptosis via regulating GPX4. BioMed Pharmacother Biomed Pharmacother. (2024) 175:116680. doi: 10.1016/j.biopha.2024.116680 38703506

[185] Bae H Song G Lee J-Y Hong T Chang M-J Lim W. Laminarin-derived from brown algae suppresses the growth of ovarian cancer cells via mitochondrial dysfunction and ER stress. Mar Drugs. (2020) 18:152. doi: 10.3390/md18030152 32182828 PMC7143329

[186] Zhou J Wang Y Fu Y Lin Z Lin H Lv G. Chelerythrine induces apoptosis and ferroptosis through Nrf2 in ovarian cancer cells. Cell Mol Biol Noisy–Gd Fr. (2024) 70:174–81. doi: 10.14715/cmb/2024.70.3.26 38650145

[187] Li B Gao Y Rankin GO Rojanasakul Y Cutler SJ Tu Y. Chaetoglobosin K induces apoptosis and G2 cell cycle arrest through p53-dependent pathway in cisplatin-resistant ovarian cancer cells. Cancer Lett. (2015) 356:418–33. doi: 10.1016/j.canlet.2014.09.023 PMC4351971 25304379

[188] Zhu J Zhu H Zhu Q Xu SL Xiao L Zhang MY. The roles of autophagy, ferroptosis and pyroptosis in the anti-ovarian cancer mechanism of harmine and their crosstalk. Sci Rep. (2024) 14:6504. doi: 10.1038/s41598-024-57196-7 38499622 PMC10948856

[189] Li J Feng L Yuan Y He T Zou X Su B. Inhibition of HOXC11 by artesunate induces ferroptosis and suppresses ovarian cancer progression through transcriptional regulation of the PROM2/PI3K/AKT pathway. World J Surg Oncol. (2024) 22:268. doi: 10.1186/s12957-024-03544-w 39380001 PMC11460135

[190] Yang Y-I Kim J-H Lee K-T Choi J-H. Costunolide induces apoptosis in platinum-resistant human ovarian cancer cells by generating reactive oxygen species. Gynecol Oncol. (2011) 123:588–96. doi: 10.1016/j.ygyno.2011.08.031 21945308

[191] Chan DW Yung MM Chan Y-S Xuan Y Yang H Xu D. MAP30 protein from momordica charantia is therapeutic and has synergic activity with cisplatin against ovarian cancer *in vivo* by altering metabolism and inducing ferroptosis. Pharmacol Res. (2020) 161:105157. doi: 10.1016/j.phrs.2020.105157 32814169

[192] Wu J Zhou T Wang Y Jiang Y Wang Y. Mechanisms and advances in anti-ovarian cancer with natural plants component. Mol Basel Switz. (2021) 26:5949. doi: 10.3390/molecules26195949 PMC8512305 34641493

[193] Ma B Zhong Y Chen R Zhan X Huang G Xiong Y. Tripterygium glycosides reverse chemotherapy resistance in ovarian cancer by targeting the NRF2/GPX4 signal axis to induce ferroptosis of drug-resistant human epithelial ovarian cancer cells. Biochem Biophys Res Commun. (2023) 665:178–86. doi: 10.1016/j.bbrc.2023.04.111 37163938

[194] Zhao Y Liang H Cui X. Obacunone regulates ferroptosis in ovarian cancer through the akt/p53 pathway. Naunyn Schmiedebergs Arch Pharmacol. (2024). doi: 10.1007/s00210-024-03738-9 39708098

[195] Fontana F Marzagalli M Raimondi M Zuco V Zaffaroni N Limonta P. δ-tocotrienol sensitizes and re-sensitizes ovarian cancer cells to cisplatin via induction of G1 phase cell cycle arrest and ROS/MAPK-mediated apoptosis. Cell Prolif. (2021) 54:e13111. doi: 10.1111/cpr.13111 34520051 PMC8560608

[196] Wang Z Liu M Li G-X Zhang L Ding K-Y Li S-Q. A herbal pair of scutellaria barbata D. Don and scleromitrion diffusum (willd.) R.J. wang induced ferroptosis in ovarian cancer A2780 cells via inducing heme catabolism and ferritinophagy. J Integr Med. (2024) 22. doi: 10.1016/j.joim.2024.10.001 39521705

[197] Wu Y Jia C Liu W Zhan W Chen Y Lu J. Sodium citrate targeting Ca2+/CAMKK2 pathway exhibits anti-tumor activity through inducing apoptosis and ferroptosis in ovarian cancer. J Adv Res. (2024) 65:89–104. doi: 10.1016/j.jare.2024.04.033 38724006 PMC11518946

[198] Wang X Chen J Tie H Tian W Zhao Y Qin L. Eriodictyol regulated ferroptosis, mitochondrial dysfunction, and cell viability via Nrf2/HO-1/NQO1 signaling pathway in ovarian cancer cells. J Biochem Mol Toxicol. (2023) 37:e23368. doi: 10.1002/jbt.23368 37020356

[199] Nelson KM Dahlin JL Bisson J Graham J Pauli GF Walters MA. The essential medicinal chemistry of curcumin. J Med Chem. (2017) 60:1620–37. doi: 10.1021/acs.jmedchem.6b00975 PMC5346970 28074653

[200] Rauf A Olatunde A Imran M Alhumaydhi FA Aljohani ASM Khan SA. Honokiol: A review of its pharmacological potential and therapeutic insights. Phytomed Int J Phytother Phytopharm. (2021) 90:153647. doi: 10.1016/j.phymed.2021.153647 34362632

[201] Hu Y Yu X Yang L Xue G Wei Q Han Z. Research progress on the antitumor effects of harmine. Front Oncol. (2024) 14:1382142. doi: 10.3389/fonc.2024.1382142 38590646 PMC10999596

[202] Yavuz M Şahin B Baykal AT Demircan T. Hydroquinidine displays a significant anticarcinogenic activity in breast and ovarian cancer cells via inhibiting cell-cycle and stimulating apoptosis. Turk J Biol Turk Biyol Derg. (2023) 47:44–60. doi: 10.55730/1300-0152.2640 PMC10388048 37529110

[203] Luo H Li B Li Z Cutler SJ Rankin GO Chen YC. Chaetoglobosin K inhibits tumor angiogenesis through downregulation of vascular epithelial growth factor-binding hypoxia-inducible factor 1α. Anticancer Drugs. (2013) 24:715–24. doi: 10.1097/CAD.0b013e3283627a0b PMC3826813 23695013

[204] Cutler HG Crumley FG Cox RH Cole RJ Dorner JW Springer JP. Chaetoglobosin K: a new plant growth inhibitor and toxin from diplodia macrospora. J Agric Food Chem. (1980) 28:139–42. doi: 10.1021/jf60227a011 7358926

[205] Greenshields AL Shepherd TG Hoskin DW. Contribution of reactive oxygen species to ovarian cancer cell growth arrest and killing by the anti-malarial drug artesunate. Mol Carcinog. (2017) 56:75–93. doi: 10.1002/mc.22474 26878598

[206] Koike T Takenaka M Suzuki N Ueda Y Mori M Hirayama T. Intracellular ferritin heavy chain plays the key role in artesunate-induced ferroptosis in ovarian serous carcinoma cells. J Clin Biochem Nutr. (2022) 71:34–40. doi: 10.3164/jcbn.21-82 35903602 PMC9309081

[207] Ma N Zhang M Hu J Wei Z Zhang S. Daphnetin induces ferroptosis in ovarian cancer by inhibiting NAD(P)H:Quinone oxidoreductase 1 (NQO1). Phytomed Int J Phytother Phytopharm. (2024) 132:155876. doi: 10.1016/j.phymed.2024.155876 39032284

[208] Huang T Zhao C-C Xue M Cao Y-F Chen L-K Chen J-X. Current progress and outlook for agrimonolide: A promising bioactive compound from agrimonia pilosa ledeb. Pharm Basel Switz. (2023) 16:150. doi: 10.3390/ph16020150 PMC9960068 37259301

[209] Dandawate PR Subramaniam D Padhye SB Anant S. Bitter melon: A panacea for inflammation and cancer. Chin J Nat Med. (2016) 14:81–100. doi: 10.1016/S1875-5364(16)60002-X 26968675 PMC5276711

[210] Yung MMH Ross FA Hardie DG Leung THY Zhan J Ngan HYS. Bitter melon (momordica charantia) extract inhibits tumorigenicity and overcomes cisplatin-resistance in ovarian cancer cells through targeting AMPK signaling cascade. Integr Cancer Ther. (2016) 15:376–89. doi: 10.1177/1534735415611747 PMC5689379 26487740

[211] Diao J Jia Y Dai E Liu J Kang R Tang D. Ferroptotic therapy in cancer: Benefits, side effects, and risks. Mol Cancer. (2024) 23:89. doi: 10.1186/s12943-024-01999-9 38702722 PMC11067110

[212] Hendricks JM Doubravsky CE Wehri E Li Z Roberts MA Deol KK. Identification of structurally diverse FSP1 inhibitors that sensitize cancer cells to ferroptosis. Cell Chem Biol. (2023) 30:1090–1103.e7. doi: 10.1016/j.chembiol.2023.04.007 37178691 PMC10524360

[213] Pontel LB Bueno-Costa A Morellato AE Carvalho Santos J Roué G Esteller M. Acute lymphoblastic leukemia necessitates GSH-dependent ferroptosis defenses to overcome FSP1-epigenetic silencing. Redox Biol. (2022) 55:102408. doi: 10.1016/j.redox.2022.102408 35944469 PMC9364119

[214] Doll S Freitas FP Shah R Aldrovandi M da Silva MC Ingold I. FSP1 is a glutathione-independent ferroptosis suppressor. Nature. (2019) 575:693–8. doi: 10.1038/s41586-019-1707-0 31634899

